# Supplementary material for: vtRNA2-1/nc886 Produces a Small RNA That Contributes to Its Tumor Suppression Action through the microRNA Pathway in Prostate Cancer
Source: Noncoding RNA. 2020 Feb 20;6(1):7. doi: 10.3390/ncrna6010007 (PMC7151618; doi:10.3390/ncrna6010007)
Supplement: Supplementary file 1 [file ncrna-06-00007-s001.zip › suppl/Supplementary_Figures_S1-S4.docx]

Article

Supplementary Figures.


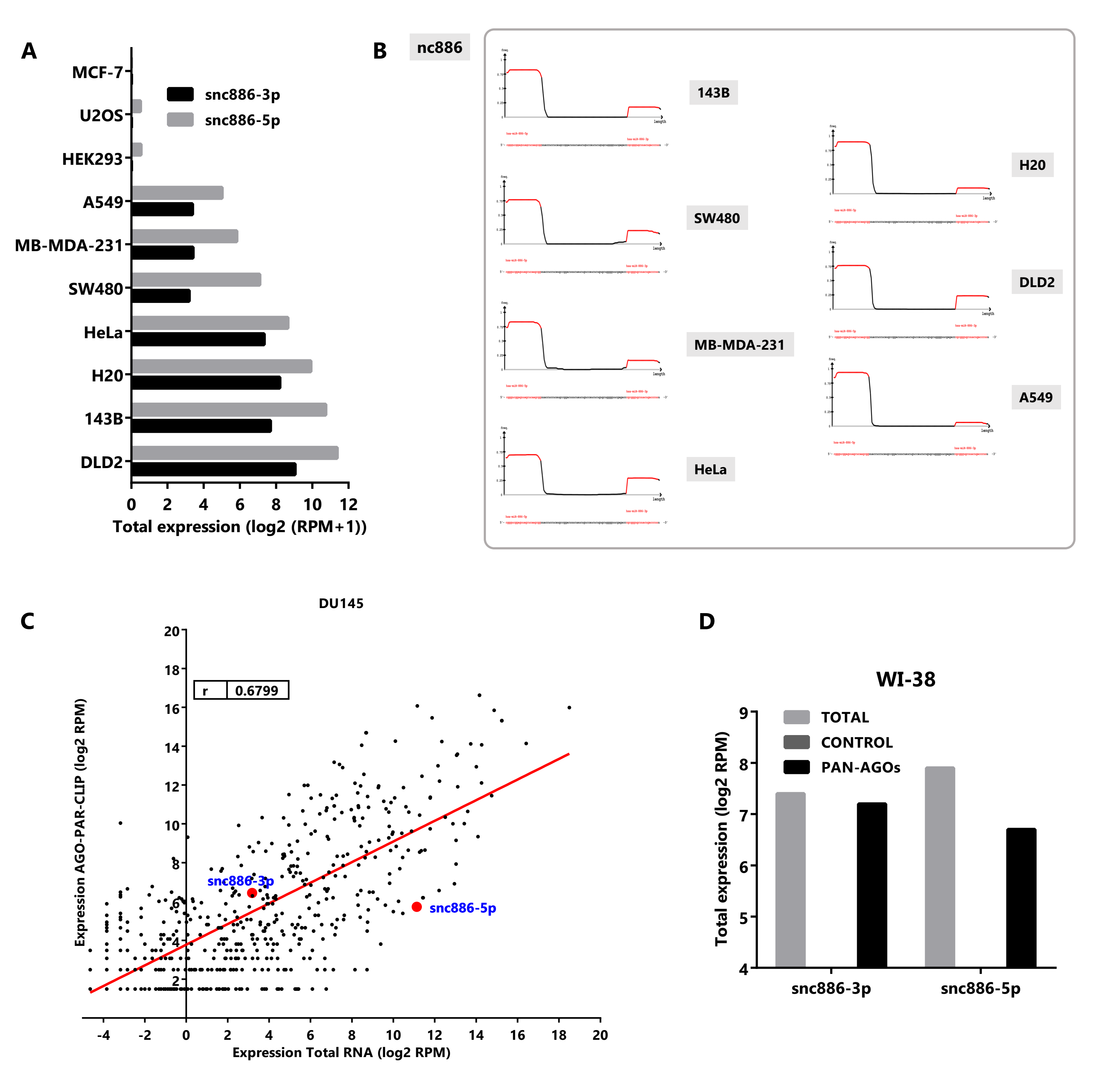


**Figure S1.** nc886 derived fragments are produced in non-prostate cell lines and exhibit microRNA features. (**a**). Normalized expression of snc886-3p (black) and 5p (gray) assessed in transcriptomic studies of small non-coding RNAs of different human cell lines. Dataset available at GEO id: GSE16579 (same used in part (**b**)). (**b**). Mapping profile of small non-coding RNAs along the nc886 sequence based on previous study. (**c**). Normalized expression of small non-coding RNAs in total cellular DU145 (TOTAL DU145) and in PAR-CLIP Argonaute RNA fraction (AGO DU145). The red dots highlight the snc886-3p and snc886-5p values in the scatterplot. Data set available at SRA id: SRP075075. (**d**). The normalized expression of snc886-3p (black) and snc886-5p (gray) in total and AGO-immunoprecipitate of WI-38 cell line. Data set available in GEO id: GSE34494.


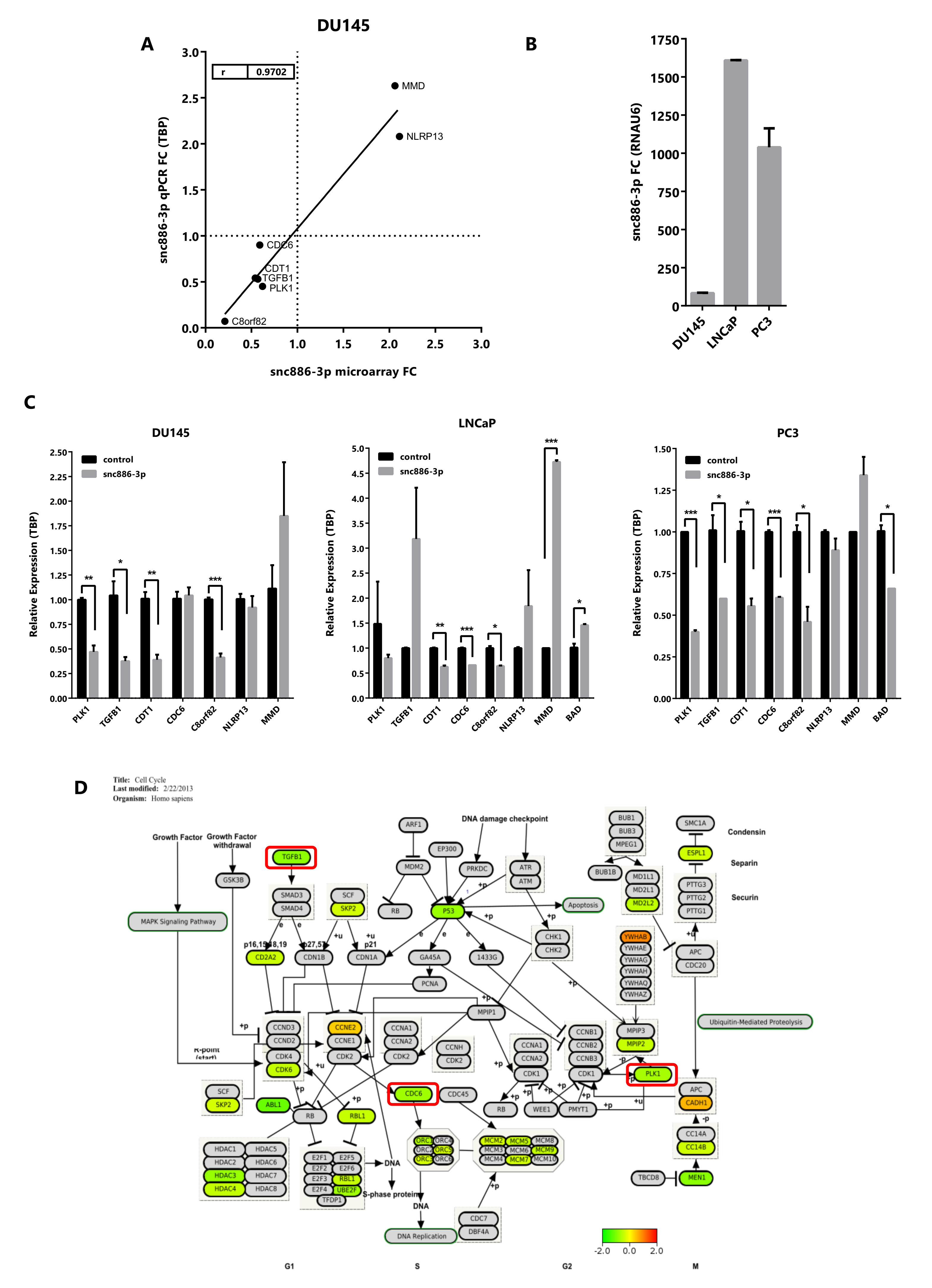


**Figure S2.** Candidate target gene expression of snc886-3p in DU145, LNCaP and DU145 (**a**). Correlation between the expression of the indicated genes caused by the overexpression of snc886-3p assessed by microarray (log2 of normalized fluorescence) and qRT-PCR (ΔCt using TBP expression as a control) in DU145 cell line. The fold change (FC) between snc886-3p mimic and control mimic is plotted. Pearson R correlation value is presented. (**b**). Expression of snc886-3p after transfection in DU145, LNCaP and PC3 with transfected with 20nM of mimic snc886-3p and negative control (Dharmacon), assessed by qRT-PCR (RNAU6 used as an internal control). (**c**). Effect of snc886-3p overexpression on 5 selected candidate direct target genes (PLK1, TGFB1, CDT1, CDC6, C8orf82) and two control genes (NLRP13 and MMD lacking site for snc886-3p and identified as DEG in DU145 array). Expression was assessed by qRT-PCR and fold change between mimic (snc886-3p) and negative control (Dharmacon) transfection in DU145, LNCaP and PC3 cell lines using TBP as a normalizer is shown. Triplicated transfections and triplicated quantifications were analyzed for each cell line; T-Test was performed, * P-value <0.05, ** P-value <0.01, *** P-value <0.001. (**d**). The DEGs modulated after the overexpression of snc886-3p identified by microarrays (fold change > 1.25 and < -1.25) are colored in green (downregulated) or red (upregulated) over a KEGG cell cycle pathway flowchart.


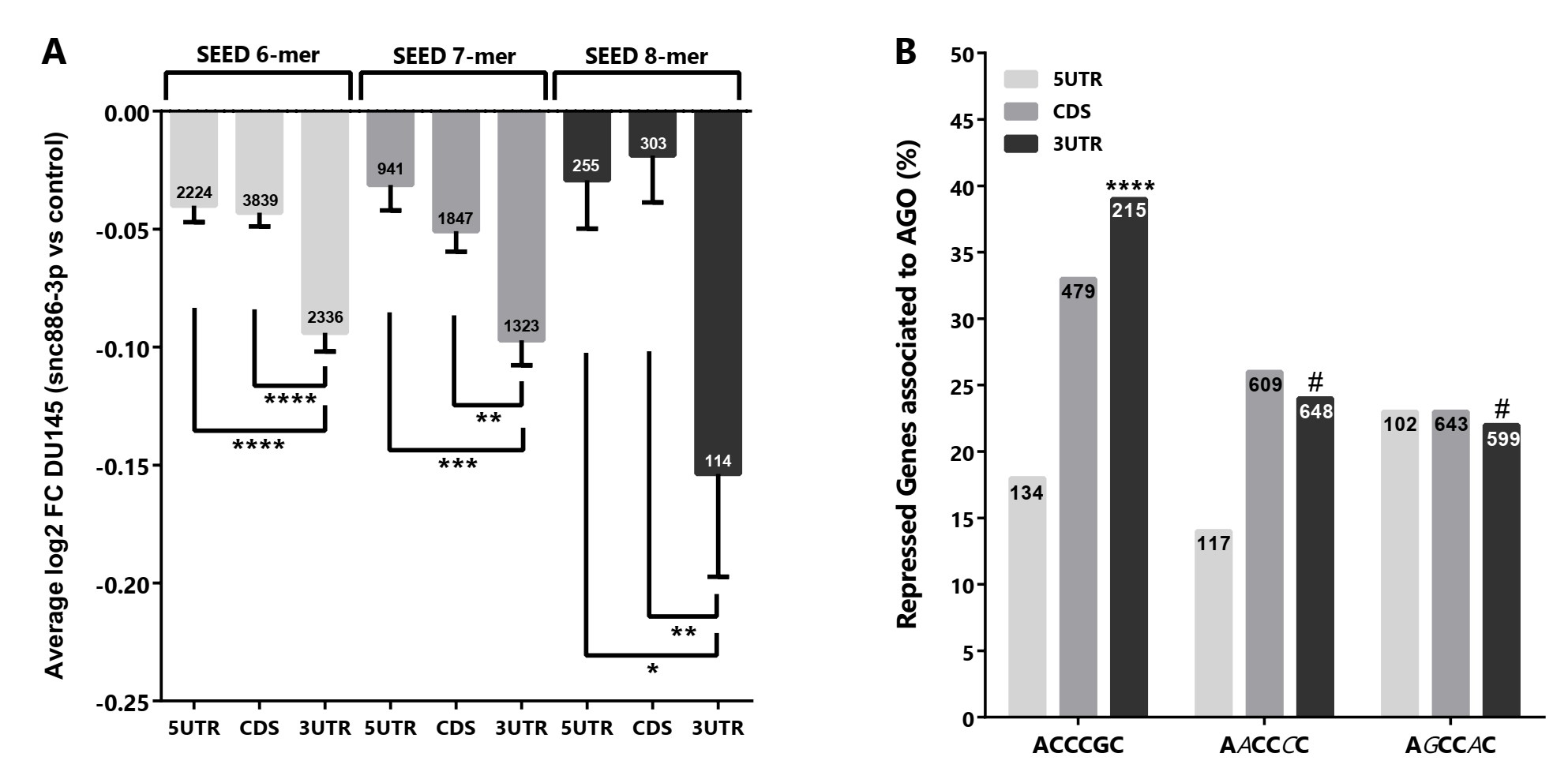


**Figure S3.** Analysis of snc886-3p microRNA-like target activity in DU145 cell line. DU145 cells were transfected with 20nM of mimic snc886-3p and negative control (Dharmacon) and cultured 48hrs. Total RNA was extracted for Affymetrix microarray global gene expression analysis. (a) Average change in the expression of all genes bearing a nucleotide motif complementary to the snc886-3p seed (5’ACCCGC3’) detected by the microarray. Seed binding site of 6-8-mer (2-7nt 5’ACCCGC3’, 2-8nt 5’cACCCGC3’, 1-8nt 5’cACCCGCg3’) were evaluated. One-way ANOVA test was used to estimate the statistical significance of the differences. (b) Percentage of genes bearing a motif complementary to the 6-mer snc886-3p seed that are downregulated in snc886-3p DU145 transfectants and present reads with this sequence motif in the AGO-bound fraction identified by PAR-CLIP experiments in DU145 cell line (Data set available at SRA id: SRP075075). The location of the motif at the 5´UTR, CDS or 3´UTR is discriminated and the number of genes in each group is shown inside the bars. Two motifs differing in 2 bases from the snc886-3p binding site were used as controls (5’A*A*CC*C*C3’ and 5’A*G*CC*A*C3’, differing in position 2 and 6 of the 6-mer scn886-3p 6-mer seed). They were verified to be not complementary to any known human microRNA binding site. Fisher’s exact test for the category “3’UTR” of 5’ACCCGC3’ yield significant different proportions for the “3’UTR” marked with # (P-value <0.0001 and Odds Ratio of 2.0 and 2.2 for 5’A*A*CCC*C*3’ and 5’A*G*CC*A*C3’ respectively).


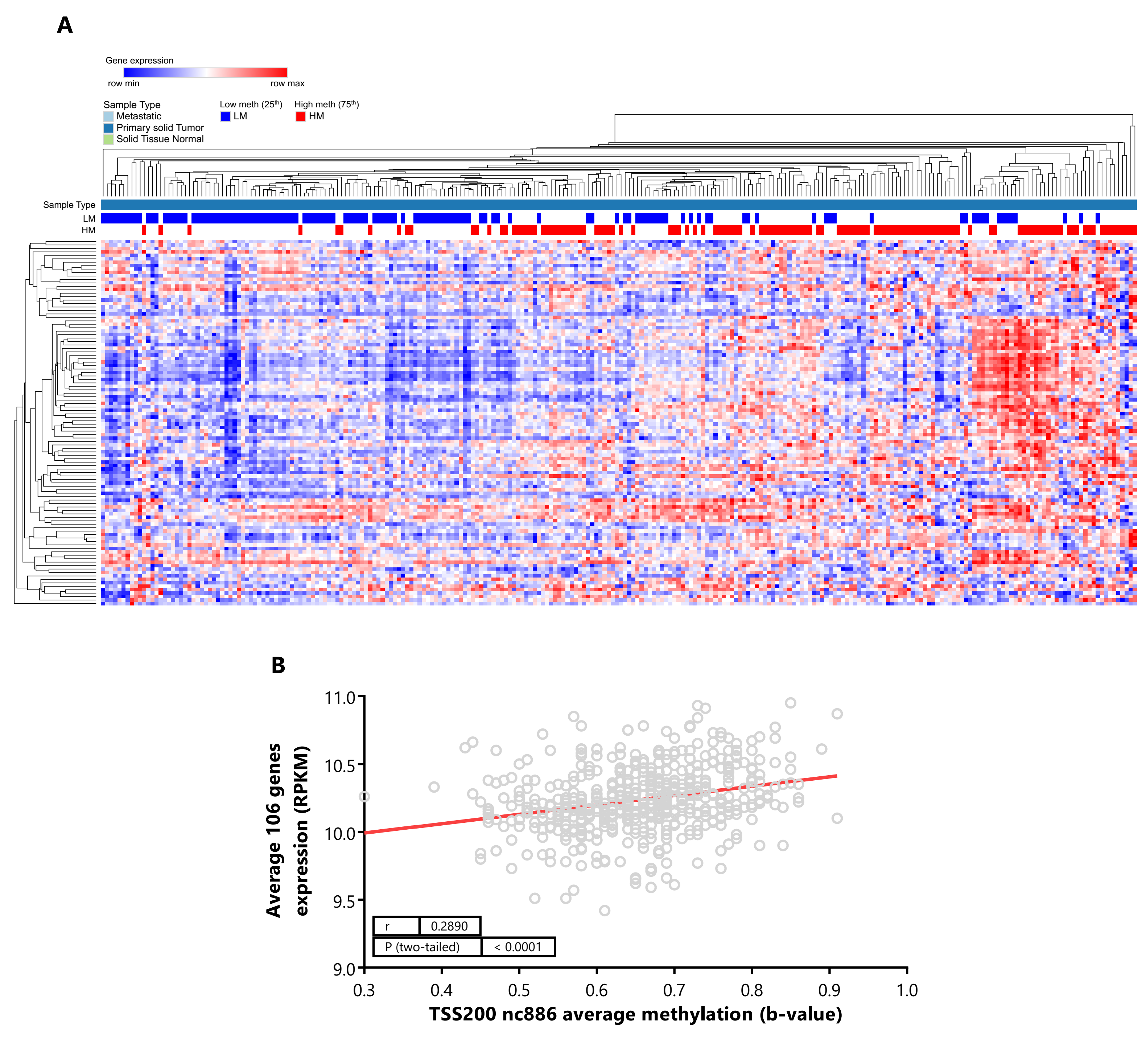


**Figure S4.** High expression of 106-snc886-3p direct targets correlates with nc886 promoter methylation in prostate tissue of PRAD-TCGA. (**a**). Heatmap of the expression of the genes of 106 direct candidate target genes of snc886-3p generated using the Spearman rank correlation algorithm clusterization with the Morpheus software and PRAD-TCGA data. The horizontal bars above the heatmap indicate the sample type and the methylation status of the nc886 TSS200nt: Percentile 25th (low methylation - high expression nc886: blue box LM) and 75th (high methylation - low expression nc886: red boxes HM). (**b**). Correlation between nc886 TSS200nt methylation and 106 snc886-3p direct candidate target gene expression in the TCGA-PRAD. Spearman R correlation and p-value are indicated.

| 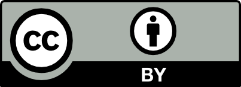 | © 2020 by the authors. Submitted for possible open access publication under the terms and conditions of the Creative Commons Attribution (CC BY) license (http://creativecommons.org/licenses/by/4.0/). |
| --- | --- |
