# Supplementary material for: vtRNA2-1/nc886 Produces a Small RNA That Contributes to Its Tumor Suppression Action through the microRNA Pathway in Prostate Cancer
Source: Noncoding RNA. 2020 Feb 20;6(1):7. doi: 10.3390/ncrna6010007 (PMC7151618; doi:10.3390/ncrna6010007)
Supplement: Supplementary file 1 [file ncrna-06-00007-s001.zip › suppl/Supplementary_Figures_S1-S6_high_quality_image.pdf]

Supplementary FIGURE 1

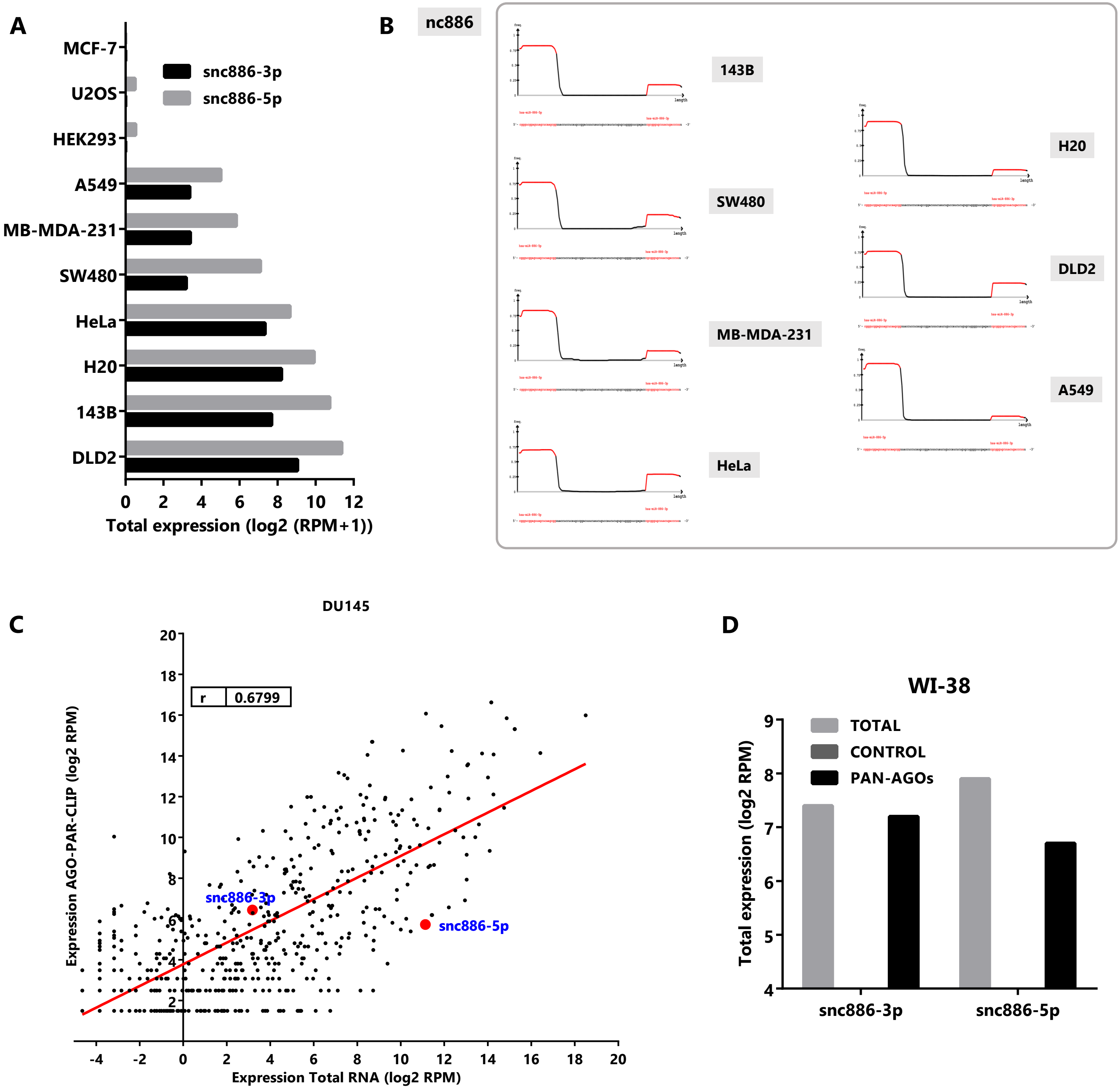

Supplementary FIGURE 2

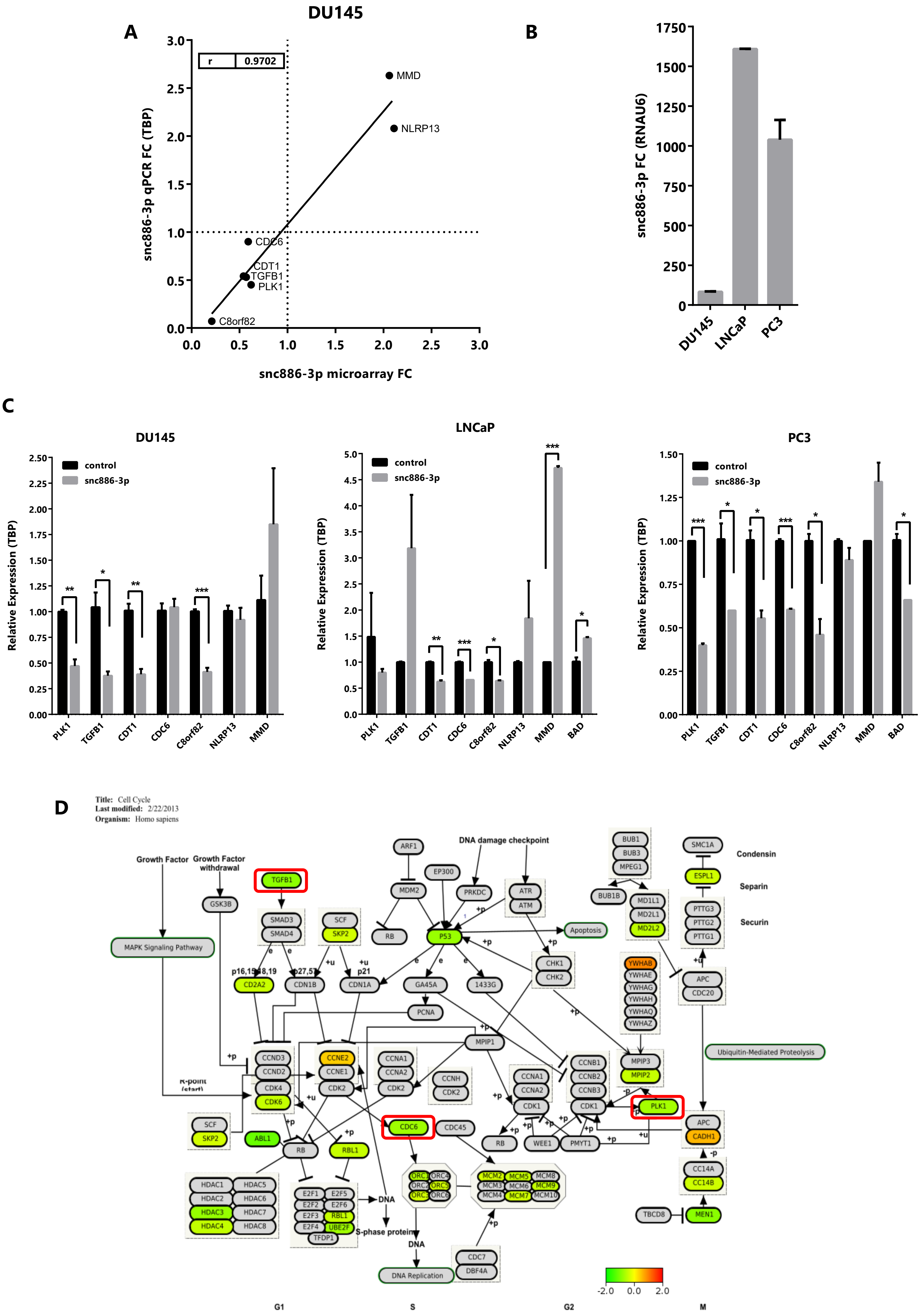

Supplementary Figure 3

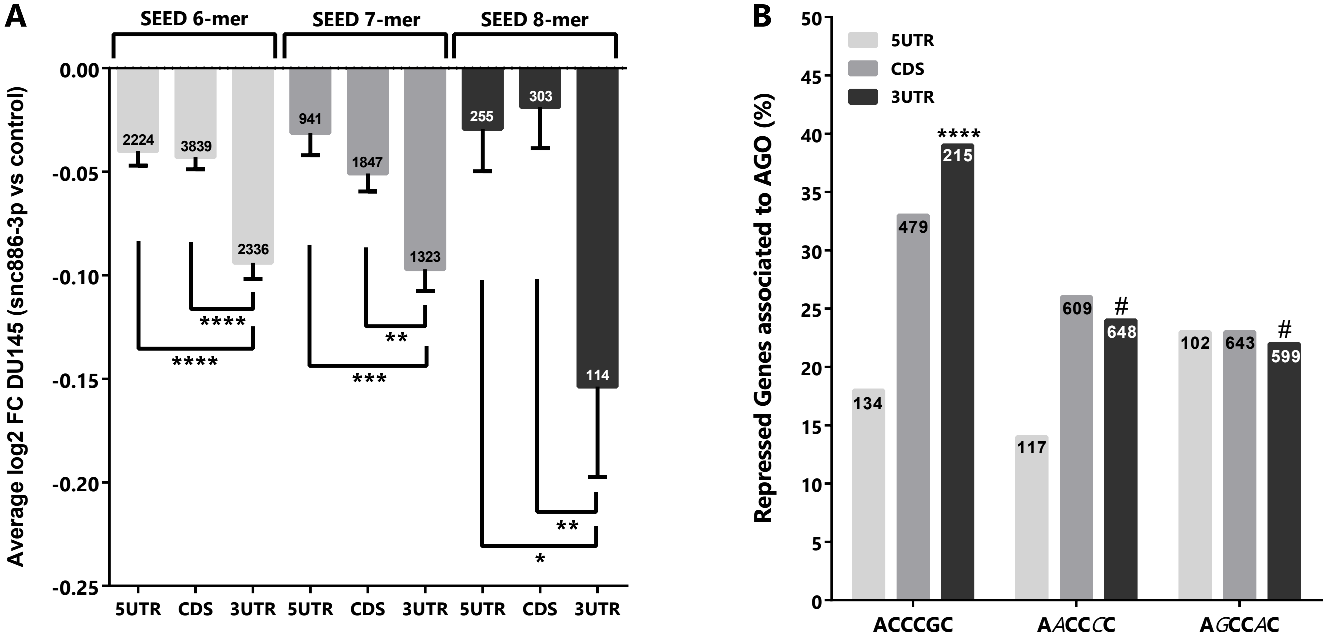

Supplementary FIGURE 4

A

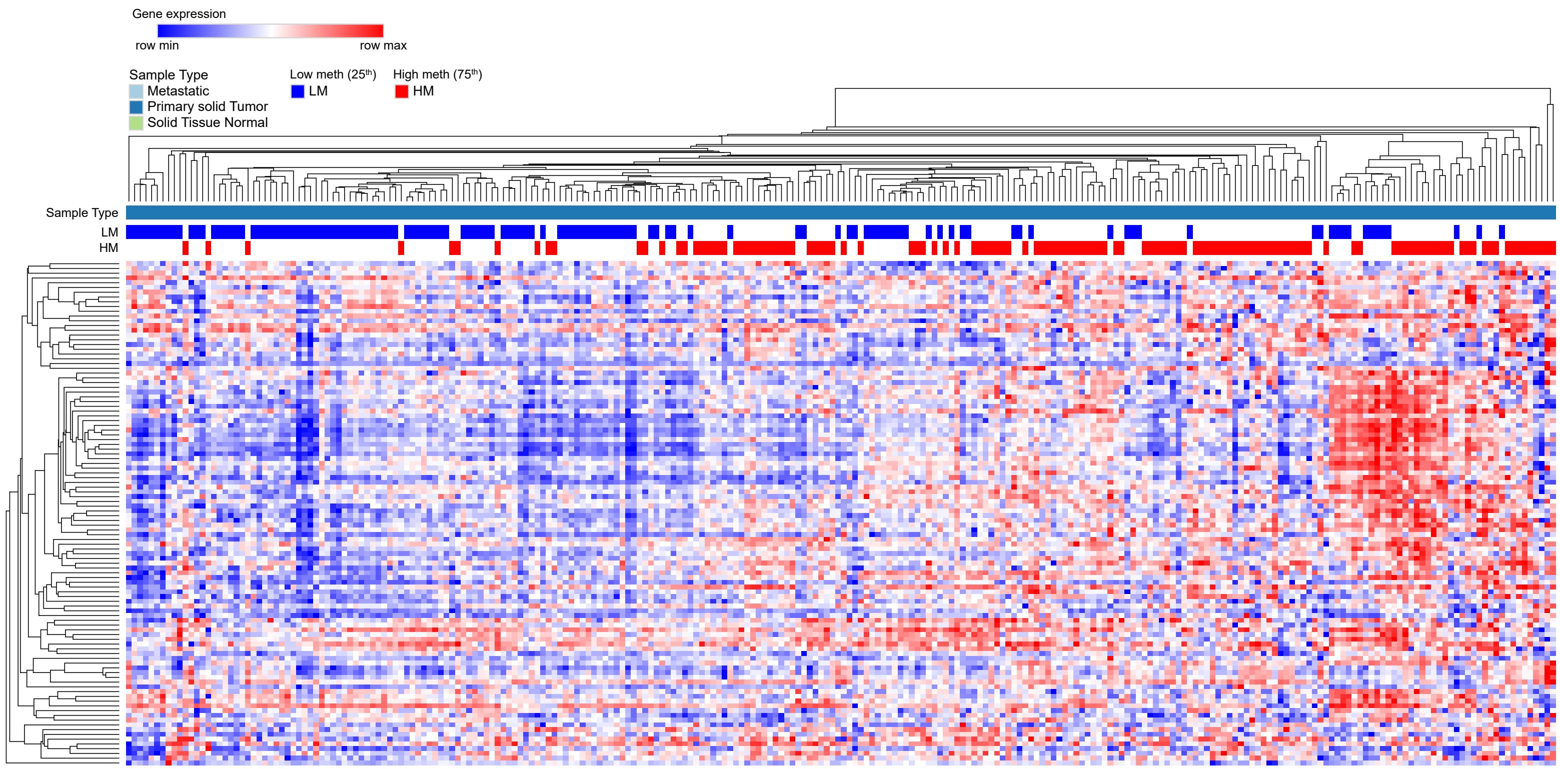

B

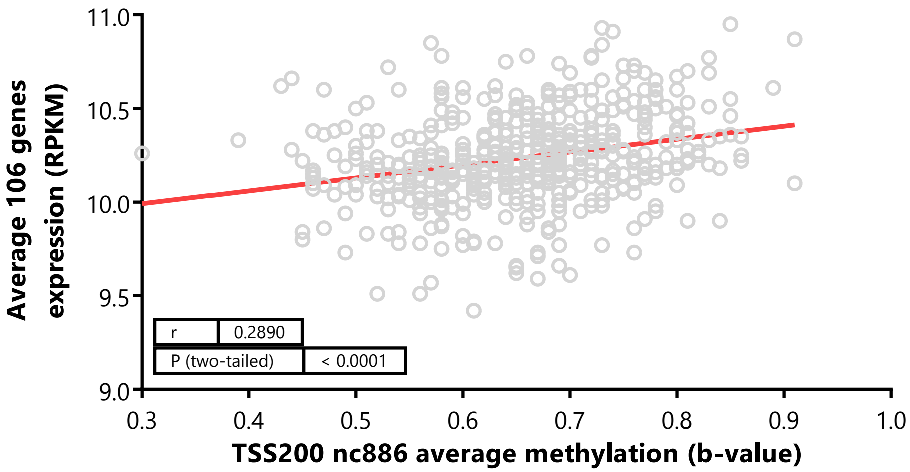

Figure S5

Non-transformed prostate cell lines (PrEc and PrSc)

miRBase precursor : hsa-mir-886
Total read count : 104
hsa-miR-886-5p read count : 36
hsa-miR-886-3p read count : 63
remaining reads : 5

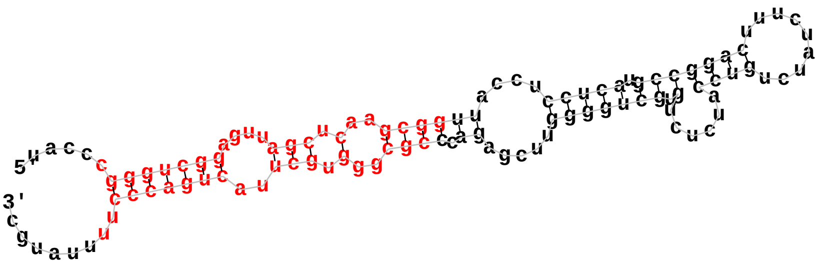

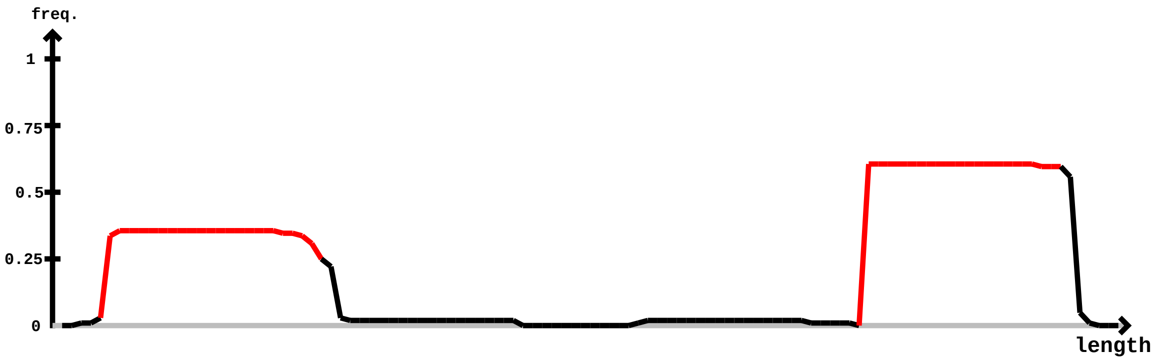


```
hsa-miR-886-5p
                                                                                  hsa-miR-886-3p
5'- uacccgggucggaguuagcucaagcgguuaccuccucaugccggacuuucuaucuguccaucucugugcuggggguucgagacccgcgggugcuuacugacccuuuuaugc   -3'    exp
    .....(((((((....(((.(..((((((....(((((.(((((((.........)))).......).))))))).....)).))))..).)))..)))))))........   reads    mm    sample
    .acccgggucggaguuagcuca.........................................................................................   1        0     seq
    ...ccgggucggaguuagcucaagc......................................................................................   2        0     seq
    ....cgggucggaguuagcucaag.......................................................................................   1        0     seq
    ....cgggucggaguuagcucaagc......................................................................................   1        0     seq
    ....cgggucggaguuagcucaagcg.....................................................................................   5        0     seq
    ....cgggucggaguuagcucaagcU.....................................................................................   1        1     seq
    ....cgggucggaguuagcucaagcgg....................................................................................   1        0     seq
    ....cgggucggaguuagcucaagcgA....................................................................................   2        1     seq
    ....cgggucggaguuagcucaagcggu...................................................................................   18       0     seq
    ....cgggucggaguuagcucaagcgAu...................................................................................   1        1     seq
    ....cgggucggaguuagcucaagcgguu..................................................................................   1        0     seq
    ....cgggAcggaguuagcucaagcgguu..................................................................................   1        1     seq
    .....gggucggaguuagcucaagcggu...................................................................................   1        0     seq
    .....gggucggaguuagcucaagcgguu..................................................................................   1        0     seq
    .............................accuccucaugccggacu................................................................   2        0     seq
    ...........................................................aucucugugcuggggguuc.................................   1        0     seq
    ............................................................ucucugugcuggggguucgagac............................   1        0     seq
    ....................................................................................cgcgggugcuuacugacA.........   1        1     seq
    ....................................................................................cgcgggugcuuacugacccuu......   4        0     seq
    ....................................................................................cgcgggugcuuacugacccCuu.....   1        1     seq
    ....................................................................................cgcgggugcuuacugacccuuA.....   3        1     seq
    ....................................................................................cgcgggugcuuacugacccuuu.....   48       0     seq
    ....................................................................................cgcgggugcuuacugacccuCu.....   1        1     seq
    ....................................................................................cgcgggugcuuacugacccuuuu....   2        0     seq
    ....................................................................................cgcgggugcuuacugacccuuuA....   2        1     seq
    ....................................................................................cgcgggugcuuacugacccuuuAa...   1        1     seq
```

Figure S6

Tumor prostate cell lines (DU145, LNCaP and PC3)

miRBase precursor : hsa-mir-886
Total read count : 66658
hsa-miR-886-5p read count : 66013
hsa-miR-886-3p read count : 278
remaining reads : 367

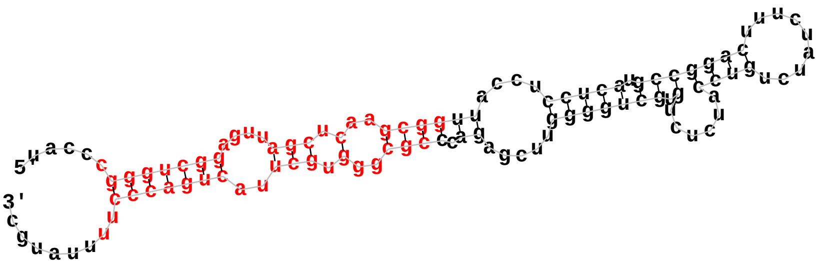

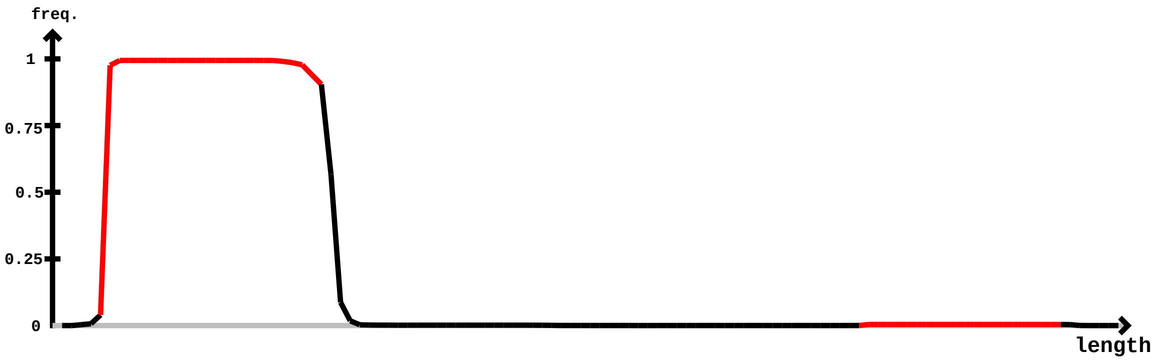

hsa-miR-886-3p
hsa-miR-886-5p

sequence	reads	mm	sample
5'- uacccgggucggaguuagcucaagcgguuaccuccucaugccggacuuucuaucuguccaucucugugcugggguucgagacccgcgggugcuuacugacccuuuuaugc -3'	exp		
.....((((((....(((.(..((((((....(((((.(((((((.........)))).......).))))))).....)).)))).).)))..)))))))........	reads	mm	sample
.acccgggucggaguuagc...	1	0	seq
.acccgggucggaCuuagcu..	1	1	seq
.acccgggucggaguuagcu..	1	0	seq
.acccgggucggaguuagcuc...	4	0	seq
.acccUggucggaguuagcuca..	1	1	seq
.acccgggucggaguuagcuca..	11	0	seq
.Ucccgggucggaguuagcuca..	1	1	seq
.aAccgggucggaguuagcuca..	1	1	seq
.acccggUucggaguuagcucaa...	1	1	seq
.Ucccgggucggaguuagcucaa...	1	1	seq
.acccgggucggaguuagcucaa...	17	0	seq
.aGccgggucggaguuagcucaa...	1	1	seq
.acccgggucggaguuagcucaCg..	1	1	seq
.acccgggucggaguuagcucaag..	29	0	seq
.acccgggucggaguuagcucaaA..	1	1	seq
.acccgggGcggaguuagcucaag..	1	1	seq
.aAccgggucggaguuagcucaag..	1	1	seq
.Gcccgggucggaguuagcucaag..	1	1	seq
.acccgggucggaguuaUcucaag..	1	1	seq
.acUcgggucggaguuagcucaag..	1	1	seq
.Ucccgggucggaguuagcucaag..	1	1	seq
.Ccccgggucggaguuagcucaagc...	1	1	seq
.acccAggucggaguuagcucaagc...	1	1	seq
.aAccgggucggaguuagcucaagc...	4	1	seq
.acGcgggucggaguuagcucaagc...	1	1	seq
.acccgggucggaguuagcucaCgc...	2	1	seq
.acccgggucggaguuagcucaagc...	94	0	seq
.acccUggucggaguuagcucaagc...	2	1	seq
.acccgggucgUaguuagcucaagc...	1	1	seq
.Ucccgggucggaguuagcucaagc...	1	1	seq
.acccgggucggaguuagcucaagA...	3	1	seq
.acccgggucggaguuagcucaagU...	1	1	seq
.acccgggucggaguuagcucaagcg..	10	0	seq
.acccgggucggaguuagcucaagcgA...	4	1	seq

```
    hsa-miR-886-5p                                                                 hsa-miR-886-3p
uacccgggucggaguuagcucaagcgguuaccuccucaugccggacuuucuaucuguccaucucugugcugggguucgagacccgcgggugcuuacugacccuuuuaugc

.aAccgggucggaguuagcucaagcgg...................................................................................  2   1   seq
.acccgggucggaguuagcucaagcgg...................................................................................  11  0   seq
.Ucccgggucggaguuagcucaagcgg...................................................................................  1   1   seq
.acUcgggucggaguuagcucaagcgg...................................................................................  1   1   seq
.acccgggucggaguuagcucaagcgU...................................................................................  4   1   seq
.acccgggucggaguuagcucaagcggu..................................................................................  4   0   seq
.acccgggucggaguuagcucaagcgguu.................................................................................  1   0   seq
.acccgggucggaguuagcucaagcgguuac...............................................................................  2   0   seq
..cUcgggucggaguuagcuc.........................................................................................  1   1   seq
..Accgggucggaguuagcuc.........................................................................................  1   1   seq
..cGcgggucggaguuagcuca........................................................................................  1   1   seq
..cccgggucggaguuagcuca........................................................................................  1   0   seq
..Accgggucggaguuagcuca........................................................................................  1   1   seq
..cccgggAcggaguuagcuca........................................................................................  1   1   seq
..cccgggucggaguuagcucaa.......................................................................................  5   0   seq
..cccgggucggaUuuagcucaa.......................................................................................  1   1   seq
..Accgggucggaguuagcucaa.......................................................................................  4   1   seq
..cccCggucggaguuagcucaag......................................................................................  1   1   seq
..cccgggucggaguuCgcucaag......................................................................................  1   1   seq
..cccgggGcggaguuagcucaag......................................................................................  1   1   seq
..cAcgggucggaguuagcucaag......................................................................................  1   1   seq
..cccgggucggaguuagcucaag......................................................................................  9   0   seq
..Accgggucggaguuagcucaag......................................................................................  3   1   seq
..cccgUgucggaguuagcucaag......................................................................................  1   1   seq
..Accgggucggaguuagcucaagc.....................................................................................  14  1   seq
..cAcgggucggaguuagcucaagc.....................................................................................  2   1   seq
..cccgggAcggaguuagcucaagc.....................................................................................  1   1   seq
..cccgggucggGguuagcucaagc.....................................................................................  1   1   seq
..Uccgggucggaguuagcucaagc.....................................................................................  2   1   seq
..cccgggCcggaguuagcucaagc.....................................................................................  2   1   seq
..cccgggucggaguuagcGcaagc.....................................................................................  1   1   seq
..cccgggucggaguuagcucaagc.....................................................................................  51  0   seq
..ccGgggucggaguuagcucaagc.....................................................................................  1   1   seq
..cAcgggucggaguuagcucaagcg....................................................................................  3   1   seq
..cccgggucggCguuagcucaagcg....................................................................................  1   1   seq
..cccgggucggaguuagcucaagcg....................................................................................  20  0   seq
..cccgUgucggaguuagcucaagcg....................................................................................  1   1   seq
..Accgggucggaguuagcucaagcg....................................................................................  9   1   seq
..cccgggucggaguuagcucaagcgU...................................................................................  1   1   seq
..cccgCgucggaguuagcucaagcgg...................................................................................  1   1   seq
..cccgggucggaguuagcucaagcgg...................................................................................  28  0   seq
..Accgggucggaguuagcucaagcgg...................................................................................  8   1   seq
..cAcgggucggaguuagcucaagcgg...................................................................................  2   1   seq
..cccCggucggaguuagcucaagcgg...................................................................................  1   1   seq
..cccgggucggagGuagcucaagcgg...................................................................................  1   1   seq
..cccgggucggaUuuagcucaagcgg...................................................................................  1   1   seq
..cccgggCcggaguuagcucaagcgg...................................................................................  1   1   seq
..Accgggucggaguuagcucaagcggu..................................................................................  5   1   seq
..cccgggucggaguuagcucaagcggu..................................................................................  17  0   seq
..cccgggucggCguuagcucaagcggu..................................................................................  1   1   seq
..cAcgggucggaguuagcucaagcggu..................................................................................  2   1   seq
..cccgggucggaguuagcucaagcgguu.................................................................................  4   0   seq
...Ucgggucggaguuagcuc.........................................................................................  1   1   seq
...ccUggucggaguuagcuc.........................................................................................  1   1   seq
...ccgggucggaguuagcuc.........................................................................................  5   0   seq
...ccCggucggaguuagcuca........................................................................................  1   1   seq
...ccgggucggaguuagcuca........................................................................................  14  0   seq
...cGggucggaguuagcuca.........................................................................................  1   1   seq
...ccUggucggaguuagcuca........................................................................................  1   1   seq
...ccgggucggaguuagcCcaa.......................................................................................  1   1   seq
...ccgggucggaguuagcucaa.......................................................................................  17  0   seq
...ccgggucggaUuuagcucaa.......................................................................................  1   1   seq
...ccUggucggaguuagcucaa.......................................................................................  1   1   seq
...ccCggucggaguuagcucaa.......................................................................................  1   1   seq
...ccCggucggaguuagcucaag......................................................................................  1   1   seq
...ccggguAggaguuagcucaag......................................................................................  1   1   seq
...ccgggAcggaguuagcucaag......................................................................................  1   1   seq
...cUgggucggaguuagcucaag......................................................................................  1   1   seq
...ccUggucggaguuagcucaag......................................................................................  1   1   seq
```

```
                                                                                   hsa-miR-886-3p
    hsa-miR-886-5p
uacccgggucggaguuagcucaagcgguuaccuccucaugccggacuuucuaucuguccaucucugugcugggguucgagacccgcgggugcuuacugacccuuuuaugc

...ccgggucUgaguuagcucaag......................................................................................    1      1      seq
...cAgggucggaguuagcucaag......................................................................................    1      1      seq
...ccgggucggaguuagcucaag......................................................................................    45     0      seq
...ccggguUggaguuagcucaag......................................................................................    1      1      seq
...ccAggucggaguuagcucaagc.....................................................................................    1      1      seq
...ccggUucggaguuagcucaagc.....................................................................................    4      1      seq
...ccggguAggaguuagcucaagc.....................................................................................    2      1      seq
...ccgggucggagAuagcucaagc.....................................................................................    1      1      seq
...ccgggucUgaguuagcucaagc.....................................................................................    1      1      seq
...ccgggucggaguuagUucaagc.....................................................................................    1      1      seq
...Acgggucggaguuagcucaagc.....................................................................................    3      1      seq
...ccgUgucggaguuagcucaagc.....................................................................................    1      1      seq
...ccggCucggaguuagcucaagc.....................................................................................    1      1      seq
...ccgggucggaguCagcucaagc.....................................................................................    1      1      seq
...ccgggucCgaguuagcucaagc.....................................................................................    1      1      seq
...ccgggucggaguuCgcucaagc.....................................................................................    1      1      seq
...cAgggucggaguuagcucaagc.....................................................................................    4      1      seq
...ccUggucggaguuagcucaagc.....................................................................................    15     1      seq
...ccgggucggaguuagcucaCgc.....................................................................................    2      1      seq
...ccCggucggaguuagcucaagc.....................................................................................    2      1      seq
...ccgggucggaguuagcCcaagc.....................................................................................    1      1      seq
...ccgggucggaguuagcucaagU.....................................................................................    5      1      seq
...ccgggucggaguuagcucaagA.....................................................................................    1      1      seq
...cUgggucggaguuagcucaagc.....................................................................................    3      1      seq
...ccgggucggaguGagcucaagc.....................................................................................    1      1      seq
...cGgggucggaguuagcucaagc.....................................................................................    3      1      seq
...ccgggucggaguuagcuGaagc.....................................................................................    1      1      seq
...ccgggucggaguuagcucaagc.....................................................................................    202    0      seq
...Ucgggucggaguuagcucaagc.....................................................................................    3      1      seq
...ccgggucgCaguuagcucaagc.....................................................................................    1      1      seq
...ccgggucggagGuagcucaagc.....................................................................................    3      1      seq
...ccgggucggaguuagcucaCgcg....................................................................................    2      1      seq
...ccgggCcggaguuagcucaagcg....................................................................................    2      1      seq
...Ucgggucggaguuagcucaagcg....................................................................................    10     1      seq
...ccgggucggagCuagcucaagcg....................................................................................    1      1      seq
...ccgggucggaguuagUucaagcg....................................................................................    1      1      seq
...ccggguAggaguuagcucaagcg....................................................................................    1      1      seq
...ccgggucggaguuagcGcaagcg....................................................................................    1      1      seq
...cGgggucggaguuagcucaagcg....................................................................................    2      1      seq
...cAgggucggaguuagcucaagcg....................................................................................    5      1      seq
...cUgggucggaguuagcucaagcg....................................................................................    2      1      seq
...ccCggucggaguuagcucaagcg....................................................................................    2      1      seq
...ccgggucggaguuagcCcaagcg....................................................................................    1      1      seq
...ccgggucUgaguuagcucaagcg....................................................................................    1      1      seq
...ccgggucggaCuuagcucaagcg....................................................................................    2      1      seq
...Acgggucggaguuagcucaagcg....................................................................................    2      1      seq
...ccgggucggaguuagcucCagcg....................................................................................    1      1      seq
...ccggUucggaguuagcucaagcg....................................................................................    4      1      seq
...ccggguUggaguuagcucaagcg....................................................................................    1      1      seq
...ccUggucggaguuagcucaagcg....................................................................................    22     1      seq
...ccgggucggaguuagcucaagcA....................................................................................    10     1      seq
...ccgggucggaguuagcucaagcg....................................................................................    200    0      seq
...ccgggucggaAuuagcucaagcg....................................................................................    1      1      seq
...ccgggucggagAuagcucaagcg....................................................................................    3      1      seq
...ccgggucggaguuagcuGaagcg....................................................................................    1      1      seq
...ccAggucggaguuagcucaagcgg...................................................................................    4      1      seq
...ccgggucggaguuagcucaCgcgg...................................................................................    4      1      seq
...ccgggucggaguuagcucaagcgg...................................................................................    818    0      seq
...ccgggucggaguuagcucaGgcgg...................................................................................    1      1      seq
...ccgggucggaguuagcucaaCcgg...................................................................................    1      1      seq
...Ucgggucggaguuagcucaagcgg...................................................................................    9      1      seq
...ccgggGcggaguuagcucaagcgg...................................................................................    2      1      seq
...ccgggucggaguuaUcucaagcgg...................................................................................    3      1      seq
...cAgggucggaguuagcucaagcgg...................................................................................    12     1      seq
...ccCggucggaguuagcucaagcgg...................................................................................    14     1      seq
...ccggUucggaguuagcucaagcgg...................................................................................    12     1      seq
...ccgUgucggaguuagcucaagcgg...................................................................................    1      1      seq
...ccggguAggaguuagcucaagcgg...................................................................................    3      1      seq
...ccgggucggagAuagcucaagcgg...................................................................................    4      1      seq
```

hsa-miR-886-3p

hsa-miR-886-5p

uacccgggucggaguuagcucaagcgguuaccuccucaugccggacuuucuaucuguccaucucugugcugggguucgagacccgcgggugcuuacugacccuuuuaugc

...ccgggucggCguuagcucaagcgg	1	1	seq
...Acgggucggaguuagcucaagcgg	15	1	seq
...ccgggucggaguuagcucaagcUg	1	1	seq
...ccgAgucggaguuagcucaagcgg	1	1	seq
...ccgggucggaUuuagcucaagcgg	5	1	seq
...ccgggucggaguuGgcucaagcgg	1	1	seq
...ccgggucggagCuagcucaagcgg	6	1	seq
...ccUggucggaguuagcucaagcgg	93	1	seq
...ccgggucggaguuagAucaagcgg	1	1	seq
...ccgggucgUaguuagcucaagcgg	1	1	seq
...ccgggucggaguuagUucaagcgg	3	1	seq
...ccgggucggagGuagcucaagcgg	2	1	seq
...ccgggucggaguuagcucaagcgU	24	1	seq
...ccgggucggaCuuagcucaagcgg	1	1	seq
...ccgggucggaguuagcucaagcgC	2	1	seq
...ccgggucggaguuagcucaagcAg	1	1	seq
...ccggAucggaguuagcucaagcgg	2	1	seq
...cUgggucggaguuagcucaagcgg	2	1	seq
...ccgggucggaguuagcucaagcgA	51	1	seq
...cGgggucggaguuagcucaagcgg	7	1	seq
...ccgggucggaguuagcucCagcgg	2	1	seq
...ccggguUggaguuagcucaagcgg	5	1	seq
...ccggCucggaguuagcucaagcgg	1	1	seq
...ccgggucUgaguuagcucaagcgg	2	1	seq
...ccggguAggaguuagcucaagcggu	2	1	seq
...ccAggucggaguuagcucaagcggu	1	1	seq
...ccgggucggagAuagcucaagcggu	1	1	seq
...ccgggucggagGuagcucaagcggu	1	1	seq
...ccgggucggaguuagcucaagcggu	293	0	seq
...ccgggucggaguuagcAcaagcggu	1	1	seq
...ccgggGcggaguuagcucaagcggu	2	1	seq
...ccgggucggaguuagcuAaagcggu	1	1	seq
...ccgggucggaUuuagcucaagcggu	1	1	seq
...ccgggucggaguuagUucaagcggu	1	1	seq
...ccgggucggCguuagcucaagcggu	1	1	seq
...ccgggucggaguuagcuGaagcggu	1	1	seq
...ccgggucggaguuagcucaUgcggu	1	1	seq
...ccgggucggaguuagcucaaUcggu	1	1	seq
...cAgggucggaguuagcucaagcggu	3	1	seq
...ccgggucggaguuagcucaagcggA	10	1	seq
...ccgggCcggaguuagcucaagcggu	1	1	seq
...ccgggucggaCuuagcucaagcggu	1	1	seq
...cUgggucggaguuagcucaagcggu	1	1	seq
...ccgggucggaguuagcucaGgcggu	1	1	seq
...ccgggucggaguuagcucaagcgUu	1	1	seq
...ccgggucggaguuagcucaagcgCu	2	1	seq
...ccggUucggaguuagcucaagcggu	8	1	seq
...Acgggucggaguuagcucaagcggu	8	1	seq
...ccgggucggUguuagcucaagcggu	1	1	seq
...ccgggucggaguuaUcucaagcggu	2	1	seq
...ccUggucggaguuagcucaagcggu	25	1	seq
...cGgggucggaguuagcucaagcggu	4	1	seq
...ccgggucggaguuagcucaagcAgu	1	1	seq
...ccgggucggaguuagcucaagAggu	1	1	seq
...ccCggucggaguuagcucaagcggu	6	1	seq
...ccggguUggaguuagcucaagcggu	1	1	seq
...ccgggucggagCuagcucaagcggu	1	1	seq
...Ucgggucggaguuagcucaagcggu	3	1	seq
...cUgggucggaguuagcucaagcgguu	1	1	seq
...cAgggucggaguuagcucaagcgguu	2	1	seq
...ccgggucggaguuagcucaagcgguu	24	0	seq
...Acgggucggaguuagcucaagcgguu	1	1	seq
...ccgggucggaUuuagcucaagcgguu	1	1	seq
...ccUggucggaguuagcucaagcgguu	1	1	seq
...cGgggucggaguuagcucaagcgguu	2	1	seq
...ccgggucggaguuagcucaagcgguA	9	1	seq
...ccgggucggaguuagcucaagAgguu	2	1	seq
...ccgggucggaguuagcucaagcgguAa	2	1	seq
...ccgggucggaguuagcucaagcgguua	3	0	seq

hsa-miR-886-3p

hsa-miR-886-5p

uacccgggucggaguuagcucaagcgguuaccuccucaugccggacuuucuaucuguccaucucugugcugggguucgagacccgcgggugcuuacugacccuuuuaugc

...ccgggucggaguuagcucaagcgguuU..	8	1	seq
...ccgggucggaguuagcucaagcgguuaU...	1	1	seq
....cgggAcggaguuagcuca..	4	1	seq
....cgggucggagGuagcuca..	1	1	seq
....cgggucggaguuagAuca..	1	1	seq
....cggguCgaguuagcuca..	1	1	seq
....cgggucggUguuagcuca..	1	1	seq
....cgggGcggaguuagcuca..	10	1	seq
....cAggucggaguuagcuca..	2	1	seq
....cgUgucggaguuagcuca..	17	1	seq
....cgggucggCguuagcuca..	3	1	seq
....cgggCcggaguuagcuca..	5	1	seq
....cgCgucggaguuagcuca..	2	1	seq
....cgggucggaguuagcuca..	123	0	seq
....cgggucggaUuuagcuca..	4	1	seq
....cgggucggaCuuagcuca..	3	1	seq
....cgggucggagCuagcuca..	1	1	seq
....cgggucggaguuCgcuca..	1	1	seq
....cggCucggaguuagcuca..	2	1	seq
....Ugggucggaguuagcuca..	6	1	seq
....cgggucggaguuagcucUa...	1	1	seq
....cggguGggaguuagcucaa...	1	1	seq
....cgggAcggaguuagcucaa...	1	1	seq
....cgggucggaguuagcucaa...	175	0	seq
....cAggucggaguuagcucaa...	1	1	seq
....cgAgucggaguuagcucaa...	3	1	seq
....cgggucggaguuagcucCa...	2	1	seq
....cggCucggaguuagcucaa...	1	1	seq
....cgggucggaguuCgcucaa...	1	1	seq
....cgggucggaCuuagcucaa...	1	1	seq
....cgggucggaguuagUucaa...	2	1	seq
....Agggucggaguuagcucaa...	4	1	seq
....cgggucggaguuagcAcaa...	1	1	seq
....Ugggucggaguuagcucaa...	7	1	seq
....cgggucggagGuagcucaa...	2	1	seq
....cgggucggaguuGgcucaa...	1	1	seq
....cggguCgaguuagcucaa...	1	1	seq
....cgggucggagCuagcucaa...	2	1	seq
....cggguUggaguuagcucaa...	1	1	seq
....cgggucggaAuuagcucaa...	1	1	seq
....cUggucggaguuagcucaa...	1	1	seq
....cgggucggCguuagcucaa...	2	1	seq
....cgggucggaguuagAucaa...	1	1	seq
....cgggucggaguAagcucaa...	1	1	seq
....cgggGcggaguuagcucaa...	9	1	seq
....cgggCcggaguuagcucaa...	2	1	seq
....cgggucggagAuagcucaa...	1	1	seq
....Ggggucggaguuagcucaa...	2	1	seq
....cgUgucggaguuagcucaa...	25	1	seq
....cggguUgaguuagcucaa...	1	1	seq
....cgCgucggaguuagcucaa...	4	1	seq
....cgggucggUguuagcucaa...	1	1	seq
....cgggucggaguuagcGcaa...	4	1	seq
....cgggucggaUuuagcucaa...	8	1	seq
....cgggucgUaguuagcucaa...	2	1	seq
....cggguUggaguuagcucaag..	3	1	seq
....cgggucggaguuagcGcaag..	2	1	seq
....cgggucgUaguuagcucaag..	2	1	seq
....cgggucggaguAagcucaag..	1	1	seq
....cgggAcggaguuagcucaag..	7	1	seq
....cggCucggaguuagcucaag..	2	1	seq
....cgggucggaguuagcucaaA..	8	1	seq
....cgggucggaguuagcAcaag..	2	1	seq
....cgggucggaguuagcCcaag..	1	1	seq
....cgggucggagCuagcucaag..	2	1	seq
....cgggucggaguuagcucGag..	1	1	seq
....cgggucggaguuagcucaCg..	1	1	seq
....cgggucggaguuagUucaag..	3	1	seq
....cgggucggaguuCgcucaag..	4	1	seq

```
                                                                                 hsa-miR-886-3p
    hsa-miR-886-5p
uacccgggucggaguuagcucaagcgguuaccuccucaugccggacuuucuaucuguccaucucugugcugggguucgagacccgcgggugcuuacugacccuuuuaugc

....cgggucgCaguuagcucaag......................................................................................    1      1      seq
....cgggucggaUuuagcucaag......................................................................................    1      1      seq
....cgggucAgaguuagcucaag......................................................................................    2      1      seq
....cgggucUgaguuagcucaag......................................................................................    2      1      seq
....cgggucggaguuaUcucaag......................................................................................    1      1      seq
....cgggucggaguGagcucaag......................................................................................    1      1      seq
....cgggucggaguuagAucaag......................................................................................    2      1      seq
....Ugggucggaguuagcucaag......................................................................................    10     1      seq
....Agggucggaguuagcucaag......................................................................................    5      1      seq
....cgggucggaCuuagcucaag......................................................................................    1      1      seq
....cgggucggaguuagcucaaU......................................................................................    4      1      seq
....cgggucggCguuagcucaag......................................................................................    4      1      seq
....cgggCcggaguuagcucaag......................................................................................    8      1      seq
....cgggucggaguCagcucaag......................................................................................    1      1      seq
....cgggucggUguuagcucaag......................................................................................    1      1      seq
....cAggucggaguuagcucaag......................................................................................    4      1      seq
....cgAgucggaguuagcucaag......................................................................................    7      1      seq
....cgggucggaAuuagcucaag......................................................................................    1      1      seq
....cgCgucggaguuagcucaag......................................................................................    4      1      seq
....cgggucggagGuagcucaag......................................................................................    5      1      seq
....cgggucggaguuagcuAaag......................................................................................    2      1      seq
....cgggGcggaguuagcucaag......................................................................................    21     1      seq
....cgggucggaguuagcucaag......................................................................................    259    0      seq
....cggguGggaguuagcucaag......................................................................................    1      1      seq
....cCggucggaguuagcucaag......................................................................................    1      1      seq
....cgUgucggaguuagcucaag......................................................................................    37     1      seq
....cgggucCgaguuagcucaag......................................................................................    1      1      seq
....cgggucgCaguuagcucaagc.....................................................................................    1      1      seq
....cgUgucggaguuagcucaagc.....................................................................................    155    1      seq
....cgggucggaguuagcucaagc.....................................................................................    1442   0      seq
....Ggggucggaguuagcucaagc.....................................................................................    2      1      seq
....cggUucggaguuagcucaagc.....................................................................................    2      1      seq
....cgggucggaguuagcucCagc.....................................................................................    7      1      seq
....cgggCcggaguuagcucaagc.....................................................................................    39     1      seq
....Agggucggaguuagcucaagc.....................................................................................    14     1      seq
....cggCucggaguuagcucaagc.....................................................................................    5      1      seq
....cgggucggaguuagcuAaagc.....................................................................................    2      1      seq
....cgggucggaguuagcCcaagc.....................................................................................    2      1      seq
....cggguUggaguuagcucaagc.....................................................................................    8      1      seq
....cgggGcggaguuagcucaagc.....................................................................................    52     1      seq
....cgggucggaguuagcucaaCc.....................................................................................    2      1      seq
....cggAucggaguuagcucaagc.....................................................................................    2      1      seq
....cgggucggaguuagAucaagc.....................................................................................    1      1      seq
....cgggucggaguuCgcucaagc.....................................................................................    13     1      seq
....cgggucggagGuagcucaagc.....................................................................................    7      1      seq
....cggguGggaguuagcucaagc.....................................................................................    9      1      seq
....cgggucggaguuUgcucaagc.....................................................................................    1      1      seq
....cgggucggaguuaUcucaagc.....................................................................................    6      1      seq
....cggguAggaguuagcucaagc.....................................................................................    1      1      seq
....cgggucggaguCagcucaagc.....................................................................................    3      1      seq
....cgggucAgaguuagcucaagc.....................................................................................    2      1      seq
....cgggucggaguuagcuUaagc.....................................................................................    1      1      seq
....cAggucggaguuagcucaagc.....................................................................................    6      1      seq
....cgggucggaguuagcucaaUc.....................................................................................    1      1      seq
....cgggucggaguAagcucaagc.....................................................................................    3      1      seq
....cgggucggaguuaCcucaagc.....................................................................................    1      1      seq
....cgggucgUaguuagcucaagc.....................................................................................    3      1      seq
....cgggucggaguuGgcucaagc.....................................................................................    3      1      seq
....cgAgucggaguuagcucaagc.....................................................................................    11     1      seq
....cgggucgAaguuagcucaagc.....................................................................................    4      1      seq
....cgggucggUguuagcucaagc.....................................................................................    18     1      seq
....cgggucggaguuagcucaagA.....................................................................................    26     1      seq
....cgggucggagCuagcucaagc.....................................................................................    7      1      seq
....Ugggucggaguuagcucaagc.....................................................................................    45     1      seq
....cgggucggaguuagcucaCgc.....................................................................................    3      1      seq
....cgggucggaCuuagcucaagc.....................................................................................    1      1      seq
....cUggucggaguuagcucaagc.....................................................................................    4      1      seq
....cgggucggaguuagcucaagU.....................................................................................    8      1      seq
....cgggAcggaguuagcucaagc.....................................................................................    14     1      seq
```

```
                                                                                    hsa-miR-886-3p
    hsa-miR-886-5p
uacccgggucggaguuagcucaagcgguuaccuccucaugccggacuuucuaucuguccaucucugugcuggggguucgagacccgcgggugcuuacugacccuuuuaugc

....cgggucggaguuagcGcaagc......................................................................................   3          1          seq
....cgCgucggaguuagcucaagc......................................................................................   16         1          seq
....cgggucUgaguuagcucaagc......................................................................................   12         1          seq
....cgggucggCguuagcucaagc......................................................................................   13         1          seq
....cgggucggaguGagcucaagc......................................................................................   8          1          seq
....cgggucggaguuagcucaUgc......................................................................................   1          1          seq
....cgggucggaAuuagcucaagc......................................................................................   1          1          seq
....cgggucggaguuagcucaagG......................................................................................   1          1          seq
....cggguAggaguuagcucaagcg.....................................................................................   3          1          seq
....cgggucggagCuagcucaagcg.....................................................................................   15         1          seq
....cgggucggaAuuagcucaagcg.....................................................................................   3          1          seq
....cggguUggaguuagcucaagcg.....................................................................................   1          1          seq
....cgggucggaguuagcucaGgcg.....................................................................................   1          1          seq
....cggguGggaguuagcucaagcg.....................................................................................   2          1          seq
....cgggucgUaguuagcucaagcg.....................................................................................   4          1          seq
....cgggucggaguuaAcucaagcg.....................................................................................   1          1          seq
....cgAgucggaguuagcucaagcg.....................................................................................   11         1          seq
....cUggucggaguuagcucaagcg.....................................................................................   3          1          seq
....cgggAcggaguuagcucaagcg.....................................................................................   11         1          seq
....cgggucggagGuagcucaagcg.....................................................................................   4          1          seq
....cgggucggaguuagcucaCgcg.....................................................................................   8          1          seq
....cgggGcggaguuagcucaagcg.....................................................................................   45         1          seq
....cgggucggaguuUgcucaagcg.....................................................................................   2          1          seq
....cgggucggaguuagcCcaagcg.....................................................................................   1          1          seq
....cgggCcggaguuagcucaagcg.....................................................................................   38         1          seq
....cgggucggaguuaUcucaagcg.....................................................................................   5          1          seq
....cgggucggaguuagcuUaagcg.....................................................................................   3          1          seq
....cgggucggaguuGgcucaagcg.....................................................................................   6          1          seq
....cgggucgAaguuagcucaagcg.....................................................................................   3          1          seq
....cgggucggaguGagcucaagcg.....................................................................................   2          1          seq
....cgggucggGguuagcucaagcg.....................................................................................   1          1          seq
....cgggucggaguuagcucCagcg.....................................................................................   14         1          seq
....cgggucggaguuagcucaagAg.....................................................................................   4          1          seq
....cgggucggaguuagcucaagcA.....................................................................................   63         1          seq
....cgggucggaguuagcucaaAcg.....................................................................................   2          1          seq
....Agggucggaguuagcucaagcg.....................................................................................   10         1          seq
....cgggucggaguuagcucaagcg.....................................................................................   1469       0          seq
....cgggucggaguuagcGcaagcg.....................................................................................   4          1          seq
....Ggggucggaguuagcucaagcg.....................................................................................   3          1          seq
....cgggucggCguuagcucaagcg.....................................................................................   12         1          seq
....cgggucggaguAagcucaagcg.....................................................................................   5          1          seq
....cgggucggaguuagcucaagUg.....................................................................................   2          1          seq
....cgggucggUguuagcucaagcg.....................................................................................   26         1          seq
....cggAucggaguuagcucaagcg.....................................................................................   1          1          seq
....Ugggucggaguuagcucaagcg.....................................................................................   42         1          seq
....cggUucggaguuagcucaagcg.....................................................................................   4          1          seq
....cgggucggaUuuagcucaagcg.....................................................................................   3          1          seq
....cgggucggaguuagAucaagcg.....................................................................................   2          1          seq
....cgggucUgaguuagcucaagcg.....................................................................................   18         1          seq
....cgCgucggaguuagcucaagcg.....................................................................................   28         1          seq
....cgggucggaguuagcucaagcU.....................................................................................   4          1          seq
....cgggucggaguuagcuAaagcg.....................................................................................   3          1          seq
....cgggucggaguuagcAcaagcg.....................................................................................   4          1          seq
....cgggucggagAuagcucaagcg.....................................................................................   4          1          seq
....cgggucggaguuagcucaUgcg.....................................................................................   4          1          seq
....cgggucggaguuCgcucaagcg.....................................................................................   17         1          seq
....cCggucggaguuagcucaagcg.....................................................................................   2          1          seq
....cggCucggaguuagcucaagcg.....................................................................................   7          1          seq
....cgUgucggaguuagcucaagcg.....................................................................................   116        1          seq
....cgggucCgaguuagcucaagcg.....................................................................................   2          1          seq
....cgggucggaguuagcucaagcC.....................................................................................   1          1          seq
....cgggucggaguCagcucaagcg.....................................................................................   6          1          seq
....cgggucggaguuagcucaaUcg.....................................................................................   4          1          seq
....cAggucggaguuagcucaagcg.....................................................................................   5          1          seq
....cgggucggaCuuagcucaagcg.....................................................................................   5          1          seq
....cgggucAgaguuagcucaagcg.....................................................................................   6          1          seq
....cgggucggaguuagAucaagcgg....................................................................................   1          1          seq
....cgggucggaguuagcuUaagcgg....................................................................................   14         1          seq
....cgggucggaguuagcucaGgcgg....................................................................................   1          1          seq
```

```
                                                                                    hsa-miR-886-3p
    hsa-miR-886-5p
uacccgggucggaguuagcucaagcgguuaccuccucaugccggacuuucuaucuguccaucucugugcuggggguucgagacccgcgggugcuuacugacccuuuuaugc

....Agggucggaguuagcucaagcgg....................................................................................   74      1   seq
....cgggCcggaguuagcucaagcgg....................................................................................   194     1   seq
....cgUgucggaguuagcucaagcgg....................................................................................   1384    1   seq
....cgggucggaguuagcCcaagcgg....................................................................................   27      1   seq
....cgggucggaguuagcGcaagcgg....................................................................................   53      1   seq
....cgggucggaguuagcucaaCcgg....................................................................................   9       1   seq
....cgggucggaguuagcucaagcgA....................................................................................   714     1   seq
....cgggucggaguuagUucaagcgg....................................................................................   5       1   seq
....cgggucggaguGagcucaagcgg....................................................................................   55      1   seq
....cgggucggaguuagcucaaUcgg....................................................................................   13      1   seq
....cggCucggaguuagcucaagcgg....................................................................................   38      1   seq
....cgggucggaguuagcucaagcgg....................................................................................   16377   0   seq
....cgggucggaguuagcucaagAgg....................................................................................   18      1   seq
....cgAgucggaguuagcucaagcgg....................................................................................   84      1   seq
....cAggucggaguuagcucaagcgg....................................................................................   58      1   seq
....cgggucggUguuagcucaagcgg....................................................................................   44      1   seq
....cCggucggaguuagcucaagcgg....................................................................................   17      1   seq
....cgggucgCaguuagcucaagcgg....................................................................................   6       1   seq
....cgggucggaUuuagcucaagcgg....................................................................................   26      1   seq
....cgggucgUaguuagcucaagcgg....................................................................................   21      1   seq
....cgggucggaguuaUcucaagcgg....................................................................................   12      1   seq
....cgggucggaguuagcuAaagcgg....................................................................................   3       1   seq
....cgggucggaguuagGucaagcgg....................................................................................   3       1   seq
....cgggucggaguuUgcucaagcgg....................................................................................   3       1   seq
....cgggucggaguuagcucaagcgU....................................................................................   198     1   seq
....cgggucggagAuagcucaagcgg....................................................................................   4       1   seq
....cggAucggaguuagcucaagcgg....................................................................................   21      1   seq
....cgggucggaguuaAcucaagcgg....................................................................................   4       1   seq
....cgggucggaguuagcucaCgcgg....................................................................................   34      1   seq
....cgggucggaAuuagcucaagcgg....................................................................................   12      1   seq
....Ugggucggaguuagcucaagcgg....................................................................................   287     1   seq
....cgggucggGguuagcucaagcgg....................................................................................   4       1   seq
....cgggucggagGuagcucaagcgg....................................................................................   53      1   seq
....cgggucggaCuuagcucaagcgg....................................................................................   6       1   seq
....cgggucggaguuagcucGagcgg....................................................................................   12      1   seq
....cgggAcggaguuagcucaagcgg....................................................................................   76      1   seq
....cggguGggaguuagcucaagcgg....................................................................................   11      1   seq
....cgggucggaguuagcucUagcgg....................................................................................   4       1   seq
....cgggucggaguuagcucaagcgC....................................................................................   33      1   seq
....cgggucggaguuagcucaagcCg....................................................................................   9       1   seq
....cgCgucggaguuagcucaagcgg....................................................................................   166     1   seq
....cgggucggaguAagcucaagcgg....................................................................................   32      1   seq
....cgggucgAaguuagcucaagcgg....................................................................................   13      1   seq
....cgggGcggaguuagcucaagcgg....................................................................................   509     1   seq
....cgggucggaguuCgcucaagcgg....................................................................................   60      1   seq
....cgggucggaguuagcucaagcUg....................................................................................   7       1   seq
....cgggucggaguuagcucaUgcgg....................................................................................   16      1   seq
....Ggggucggaguuagcucaagcgg....................................................................................   9       1   seq
....cggguCAgaguuagcucaagcgg....................................................................................   6       1   seq
....cgggucggaguuGgcucaagcgg....................................................................................   14      1   seq
....cgggucggagCuagcucaagcgg....................................................................................   42      1   seq
....cgggucCgaguuagcucaagcgg....................................................................................   8       1   seq
....cggguUggaguuagcucaagcgg....................................................................................   28      1   seq
....cgggucggaguCagcucaagcgg....................................................................................   39      1   seq
....cgggucggaguuagcAcaagcgg....................................................................................   14      1   seq
....cgggucUgaguuagcucaagcgg....................................................................................   32      1   seq
....cgggucggaguuagcucaagUgg....................................................................................   5       1   seq
....cgggucggaguuagcucaagGgg....................................................................................   8       1   seq
....cgggucggaguuaCcucaagcgg....................................................................................   2       1   seq
....cggUucggaguuagcucaagcgg....................................................................................   10      1   seq
....cUggucggaguuagcucaagcgg....................................................................................   11      1   seq
....cgggucggCguuagcucaagcgg....................................................................................   52      1   seq
....cgggucggaguuagcucCagcgg....................................................................................   30      1   seq
....cgggucggaguuagcucaagcAg....................................................................................   11      1   seq
....cggguAggaguuagcucaagcgg....................................................................................   2       1   seq
....cgggucggaguuagcucaaAcgg....................................................................................   2       1   seq
....cgggucggaguuagcucaaUcggu...................................................................................   31      1   seq
....cgggucggaguuagAucaagcggu...................................................................................   12      1   seq
....cggguAggaguuagcucaagcggu...................................................................................   6       1   seq
```

```
                                                                                 hsa-miR-886-3p
    hsa-miR-886-5p
uacccgggucggaguuagcucaagcgguuaccuccucaugccggacuuucuaucuguccaucucugugcugggguucgagacccgcgggugcuuacugacccuuuuaugc

....cgggucggaguuagcucCagcggu..................................................................................   34     1   seq
....cgggucggaCuuagcucaagcggu..................................................................................   17     1   seq
....cgggucggaguuCgcucaagcggu..................................................................................   85     1   seq
....cgggucggaguAagcucaagcggu..................................................................................   54     1   seq
....cgggucggCguuagcucaagcggu..................................................................................   91     1   seq
....cgggucggaguuaCcucaagcggu..................................................................................   1      1   seq
....cgggucggaguuagcucaagAggu..................................................................................   23     1   seq
....Ugggucggaguuagcucaagcggu..................................................................................   391    1   seq
....cgggucggaguCagcucaagcggu..................................................................................   60     1   seq
....Ggggucggaguuagcucaagcggu..................................................................................   27     1   seq
....cgggucggaguuUgcucaagcggu..................................................................................   3      1   seq
....cgggucAgaguuagcucaagcggu..................................................................................   17     1   seq
....cggAucggaguuagcucaagcggu..................................................................................   20     1   seq
....cgggucggaguuagcucaagcgCu..................................................................................   108    1   seq
....cgggucgUaguuagcucaagcggu..................................................................................   37     1   seq
....cCggucggaguuagcucaagcggu..................................................................................   25     1   seq
....cgggucCgaguuagcucaagcggu..................................................................................   7      1   seq
....cgAgucggaguuagcucaagcggu..................................................................................   123    1   seq
....cgggucggaguuagcucaagcCgu..................................................................................   12     1   seq
....cgggucggaguuagGucaagcggu..................................................................................   9      1   seq
....cggguGggaguuagcucaagcggu..................................................................................   25     1   seq
....cggUucggaguuagcucaagcggu..................................................................................   19     1   seq
....cgggGcggaguuagcucaagcggu..................................................................................   630    1   seq
....cUggucggaguuagcucaagcggu..................................................................................   20     1   seq
....cgggucggaguuagcucaaAcggu..................................................................................   10     1   seq
....cgggucggaguuagcucGagcggu..................................................................................   25     1   seq
....cgggucggaguuagcucaagcggC..................................................................................   32     1   seq
....cgggAcggaguuagcucaagcggu..................................................................................   120    1   seq
....cgggucggagGuagcucaagcggu..................................................................................   92     1   seq
....cgggucggaguuagcucaagcggA..................................................................................   861    1   seq
....cgggucggaguuagcucaGgcggu..................................................................................   8      1   seq
....cgggucUgaguuagcucaagcggu..................................................................................   64     1   seq
....cgCgucggaguuagcucaagcggu..................................................................................   197    1   seq
....cgggucggaguuagcucaagcgAu..................................................................................   15     1   seq
....cgggucggUguuagcucaagcggu..................................................................................   65     1   seq
....cgggucgAaguuagcucaagcggu..................................................................................   15     1   seq
....cgggucggaguuagcucaagGggu..................................................................................   19     1   seq
....cgggucggagAuagcucaagcggu..................................................................................   12     1   seq
....cgggucggaguuagcucaaCcggu..................................................................................   3      1   seq
....cgggCcggaguuagcucaagcggu..................................................................................   313    1   seq
....cgggucggaguuagcucaagUggu..................................................................................   13     1   seq
....cgggucggaguGagcucaagcggu..................................................................................   69     1   seq
....cgggucggaguuagcAcaagcggu..................................................................................   10     1   seq
....cggguUggaguuagcucaagcggu..................................................................................   49     1   seq
....cgggucggaguuagcucaagcgUu..................................................................................   21     1   seq
....cgggucggaguuagcucaagcAgu..................................................................................   4      1   seq
....cgggucggagCuagcucaagcggu..................................................................................   70     1   seq
....cggCucggaguuagcucaagcggu..................................................................................   67     1   seq
....cgggucggaguuGgcucaagcggu..................................................................................   13     1   seq
....cgUgucggaguuagcucaagcggu..................................................................................   1954   1   seq
....cgggucggaguuagcuUaagcggu..................................................................................   31     1   seq
....cgggucggGguuagcucaagcggu..................................................................................   11     1   seq
....cgggucggaguuagcuGaagcggu..................................................................................   2      1   seq
....cgggucgCaguuagcucaagcggu..................................................................................   6      1   seq
....cgggucggaguuagcucaCgcggu..................................................................................   86     1   seq
....cgggucggaAuuagcucaagcggu..................................................................................   29     1   seq
....cgggucggaguuaUcucaagcggu..................................................................................   23     1   seq
....cgggucggaguuagcucUagcggu..................................................................................   6      1   seq
....cgggucggaguuagcCcaagcggu..................................................................................   23     1   seq
....cgggucggaguuagcucaagcggu..................................................................................   24432  0   seq
....cgggucggaguuagcuAaagcggu..................................................................................   6      1   seq
....cgggucggaUuuagcucaagcggu..................................................................................   30     1   seq
....cgggucggaguuagcucaagcUgu..................................................................................   15     1   seq
....cgggucggaguuagcGcaagcggu..................................................................................   64     1   seq
....cgggucggaguuaAcucaagcggu..................................................................................   5      1   seq
....Agggucggaguuagcucaagcggu..................................................................................   107    1   seq
....cgggucggaguuagUucaagcggu..................................................................................   5      1   seq
....cgggucggaguuagcucaUgcggu..................................................................................   27     1   seq
....cgggucggaguuagcucaagcggG..................................................................................   12     1   seq
```

```
                                                                                  hsa-miR-886-3p
    hsa-miR-886-5p
uacccgggucggaguuagcucaagcgguuaccuccucaugccggacuuucuaucuguccaucucugugcugggguucgagacccgcgggugcuuacugacccuuuuaugc

....cAggucggaguuagcucaagcggu..................................................................................   63    1   seq
....cgggucggaguuagUucaagcgguu.................................................................................   1     1   seq
....cgggucggaCuuagcucaagcgguu.................................................................................   1     1   seq
....cggggucCgaguuagcucaagcgguu................................................................................   2     1   seq
....cgggucggaguuagcucaagcCguu.................................................................................   3     1   seq
....cgggucggaguuagcucCagcgguu.................................................................................   2     1   seq
....cgggucggagAuagcucaagcgguu.................................................................................   1     1   seq
....cgggucggaguuagcucaagcggGu.................................................................................   3     1   seq
....cgggucggaguuagcucaagcgCuu.................................................................................   2     1   seq
....cgggucggaguuagcucaagcUguu.................................................................................   4     1   seq
....cgggucggaguuagGucaagcgguu.................................................................................   1     1   seq
....Agggucggaguuagcucaagcgguu.................................................................................   8     1   seq
....cgggucggaguuagcucaagcgguG.................................................................................   105   1   seq
....cgggucAgaguuagcucaagcgguu.................................................................................   1     1   seq
....cggAucggaguuagcucaagcgguu.................................................................................   3     1   seq
....cgCgucggaguuagcucaagcgguu.................................................................................   28    1   seq
....Ugggucggaguuagcucaagcgguu.................................................................................   43    1   seq
....cAggucggaguuagcucaagcgguu.................................................................................   5     1   seq
....cgggucggaguuagcucaagcggCu.................................................................................   5     1   seq
....cgAgucggaguuagcucaagcgguu.................................................................................   4     1   seq
....cgggucggaguuCgcucaagcgguu.................................................................................   6     1   seq
....cggCucggaguuagcucaagcgguu.................................................................................   4     1   seq
....cgggucggaguuagcucaUgcgguu.................................................................................   5     1   seq
....cgggucggaguCagcucaagcgguu.................................................................................   7     1   seq
....cgggAcggaguuagcucaagcgguu.................................................................................   14    1   seq
....cgggucggagGuagcucaagcgguu.................................................................................   11    1   seq
....cgggucggaguuagcCcaagcgguu.................................................................................   2     1   seq
....cgggucggaguuagcucaagcgguu.................................................................................   2608  0   seq
....cgUgucggaguuagcucaagcgguu.................................................................................   213   1   seq
....cgggucgUaguuagcucaagcgguu.................................................................................   3     1   seq
....cgggucggaguuagcucaagcgAuu.................................................................................   3     1   seq
....cgggucggaguAagcucaagcgguu.................................................................................   7     1   seq
....cgggucggaguuGgcucaagcgguu.................................................................................   1     1   seq
....cgggucggUguuagcucaagcgguu.................................................................................   3     1   seq
....cgggucggaguuagcucGagcgguu.................................................................................   4     1   seq
....cgggucgAaguuagcucaagcgguu.................................................................................   1     1   seq
....cgggucggaguuagcucaaUcgguu.................................................................................   7     1   seq
....cgggucggaguuagcucaaCcgguu.................................................................................   1     1   seq
....cggUucggaguuagcucaagcgguu.................................................................................   3     1   seq
....cgggucggGguuagcucaagcgguu.................................................................................   1     1   seq
....cCggucggaguuagcucaagcgguu.................................................................................   3     1   seq
....cgggucggaguuagcucaagGgguu.................................................................................   2     1   seq
....cgggucggaguuagcucaagcggAu.................................................................................   69    1   seq
....cgggucggaguuagcucaagcgUuu.................................................................................   1     1   seq
....cgggucggaguuagcAcaagcgguu.................................................................................   2     1   seq
....cgggucggaguuaUcucaagcgguu.................................................................................   1     1   seq
....cgggucggaguuagcucaCgcgguu.................................................................................   10    1   seq
....cgggucggaguGagcucaagcgguu.................................................................................   9     1   seq
....Ggggucggaguuagcucaagcgguu.................................................................................   7     1   seq
....cgggucggaguuagcGcaagcgguu.................................................................................   6     1   seq
....cgggCcggaguuagcucaagcgguu.................................................................................   35    1   seq
....cgggucggaguuagcuUaagcgguu.................................................................................   1     1   seq
....cgggucggaAuuagcucaagcgguu.................................................................................   2     1   seq
....cgggGcggaguuagcucaagcgguu.................................................................................   70    1   seq
....cgggucggaUuuagcucaagcgguu.................................................................................   2     1   seq
....cgggucggaguuagcucaagcgguC.................................................................................   8     1   seq
....cggguUggaguuagcucaagcgguu.................................................................................   5     1   seq
....cgggucggaguuagcucaaAcgguu.................................................................................   1     1   seq
....cgggucggagCuagcucaagcgguu.................................................................................   3     1   seq
....cgggucggCguuagcucaagcgguu.................................................................................   6     1   seq
....cgggucggaguuagcucaagcgguA.................................................................................   932   1   seq
....cgUgucggaguuagcucaagcgguua................................................................................   8     1   seq
....cgggCcggaguuagcucaagcgguua................................................................................   1     1   seq
....cgggGcggaguuagcucaagcgguua................................................................................   3     1   seq
....cgggucggaguuagcucaCgcgguua................................................................................   1     1   seq
....cgggucggaguuagcucaaUcgguua................................................................................   1     1   seq
....cgggucggaguuagcucaagcgguGa................................................................................   9     1   seq
....cgggucggagCuagcucaagcgguua................................................................................   1     1   seq
....Ugggucggaguuagcucaagcgguua................................................................................   1     1   seq
```

```
                                                                                 hsa-miR-886-3p
    hsa-miR-886-5p
uacccgggucggaguuagcucaagcgguuaccuccucaugccggacuuucuaucuguccaucucugugcuggggguucgagacccgcgggugcuuacugacccuuuuaugc

....cgggucggaguuagcucaagcgguuG.................................................................................  3     1     seq
....cgggucggaguuagcucaagcgguuU.................................................................................  532   1     seq
....cgggucggaguuagcucaagcgguua.................................................................................  69    0     seq
....cgggucggaguuagcucaaAcgguua.................................................................................  1     1     seq
....cggUucggaguuagcucaagcgguua.................................................................................  1     1     seq
....cgggucggaguuagcucaagcgguAa.................................................................................  299   1     seq
....cgggucggaguuagcucaagcgguAac................................................................................  1     1     seq
....cgggucggaguuagcucaagcgguuaG................................................................................  1     1     seq
....cgggucggaguuagcucaagcgguuUc................................................................................  1     1     seq
....cgggucggaguuagcucaagcgguuaA................................................................................  13    1     seq
....cgggucggaguuagcucaagcgguuaU................................................................................  28    1     seq
....cgggucggaguuagcucaagcgguuac................................................................................  2     0     seq
....cgggucggaguuagcucaagcgguuaUc...............................................................................  1     1     seq
....cgggucggaguuagcucaagcgguuacc...............................................................................  6     0     seq
....cgUgucggaguuagcucaagcgguuacc...............................................................................  2     1     seq
....cgggucUgaguuagcucaagcgguuacc...............................................................................  1     1     seq
....Ugggucggaguuagcucaagcgguuacc...............................................................................  1     1     seq
....cgggucggaguuagcucaagcgguuacA...............................................................................  2     1     seq
....cgggucggaguuagcucaagcgguuaUcu..............................................................................  1     1     seq
....cgggucggaguuagcucaagcgguuaccu..............................................................................  1     0     seq
....cgUgucggaguuagcucaagcgguuaccu..............................................................................  1     1     seq
....cgggucggaguuagcucaagcgguuaAcu..............................................................................  1     1     seq
....cgggucggaguuagcucaagcgguuaccuc.............................................................................  1     0     seq
....cgggucggaguuagcucaagcgguuaAcuc.............................................................................  21    1     seq
....cgUgucggaguuagcucaagcgguuaccuc.............................................................................  1     1     seq
.....gggucggaguuagcucaa........................................................................................  4     0     seq
.....ggAucggaguuagcucaa........................................................................................  1     1     seq
.....ggUucggaguuagcucaag.......................................................................................  2     1     seq
.....gggucggaguuagcucaag.......................................................................................  3     0     seq
.....Cggucggaguuagcucaag.......................................................................................  1     1     seq
.....gggucggaguuagcucaagc......................................................................................  12    0     seq
.....ggCucggaguuagcucaagc......................................................................................  1     1     seq
.....ggAucggaguuagcucaagc......................................................................................  1     1     seq
.....gggucggaguuCgcucaagc......................................................................................  1     1     seq
.....gggucggaguuagcucaCgc......................................................................................  1     1     seq
.....gggucggaguAagcucaagc......................................................................................  1     1     seq
.....Uggucggaguuagcucaagc......................................................................................  3     1     seq
.....ggUucggaguuagcucaagcg.....................................................................................  2     1     seq
.....Cggucggaguuagcucaagcg.....................................................................................  2     1     seq
.....ggCucggaguuagcucaagcg.....................................................................................  1     1     seq
.....gggucggaguuagcucaagcg.....................................................................................  12    0     seq
.....Aggucggaguuagcucaagcg.....................................................................................  1     1     seq
.....gggucggaguuagcucaCgcg.....................................................................................  1     1     seq
.....gggucggaguuagcucaGgcg.....................................................................................  1     1     seq
.....Uggucggaguuagcucaagcg.....................................................................................  1     1     seq
.....gggucggaguuCgcucaagcg.....................................................................................  1     1     seq
.....gggucggaguuagcucaagUg.....................................................................................  1     1     seq
.....gggucggaCuuagcucaagcg.....................................................................................  1     1     seq
.....ggCucggaguuagcucaagcgg....................................................................................  2     1     seq
.....gggucggCguuagcucaagcgg....................................................................................  2     1     seq
.....Cggucggaguuagcucaagcgg....................................................................................  1     1     seq
.....gggucggaguuagcucaagcgA....................................................................................  1     1     seq
.....gggucggaguuagcGcaagcgg....................................................................................  1     1     seq
.....ggguUggaguuagcucaagcgg....................................................................................  1     1     seq
.....gggucggaguuagcucaagcgg....................................................................................  101   0     seq
.....gggucAgaguuagcucaagcgg....................................................................................  1     1     seq
.....ggUucggaguuagcucaagcgg....................................................................................  18    1     seq
.....gggCcggaguuagcucaagcgg....................................................................................  1     1     seq
.....gggucggUguuagcucaagcgg....................................................................................  3     1     seq
.....Uggucggaguuagcucaagcgg....................................................................................  8     1     seq
.....gggucggaguCagcucaagcgg....................................................................................  1     1     seq
.....gCgucggaguuagcucaagcgg....................................................................................  1     1     seq
.....gggucggaguuagcucaagcUg....................................................................................  1     1     seq
.....gggucggaAuuagcucaagcggu...................................................................................  1     1     seq
.....Uggucggaguuagcucaagcggu...................................................................................  15    1     seq
.....ggguUggaguuagcucaagcggu...................................................................................  3     1     seq
.....gggucggaguuagcucaGgcggu...................................................................................  1     1     seq
.....gggucgUaguuagcucaagcggu...................................................................................  1     1     seq
.....ggAucggaguuagcucaagcggu...................................................................................  5     1     seq
```

```
                                                                                 hsa-miR-886-3p
    hsa-miR-886-5p
uacccgggucggaguuagcucaagcgguuaccuccucaugccggacuuucuaucuguccaucucugugcugggguucgagacccgcgggugcuuacugacccuuuuaugc

.....gggucggaguuagcucaagcggu..................................................................................   503   0   seq
.....Cggucggaguuagcucaagcggu..................................................................................   8     1   seq
.....gggucggaguuagcucaaUcggu..................................................................................   3     1   seq
.....gggucggaguuagcucaCgcggu..................................................................................   5     1   seq
.....gggucggagGuagcucaagcggu..................................................................................   1     1   seq
.....gAgucggaguuagcucaagcggu..................................................................................   1     1   seq
.....gggucggaguuagcucaagcgUu..................................................................................   1     1   seq
.....Aggucggaguuagcucaagcggu..................................................................................   4     1   seq
.....gggucggaguuagcucaagcgCu..................................................................................   1     1   seq
.....gggucggaguuCgcucaagcggu..................................................................................   3     1   seq
.....gggucggaguuagcuUaagcggu..................................................................................   1     1   seq
.....gggucggUguuagcucaagcggu..................................................................................   2     1   seq
.....gggucggaCuuagcucaagcggu..................................................................................   1     1   seq
.....gggucggaguuGgcucaagcggu..................................................................................   1     1   seq
.....gggucggaguuagcucaagcggC..................................................................................   1     1   seq
.....ggCucggaguuagcucaagcggu..................................................................................   17    1   seq
.....gggucggaguCagcucaagcggu..................................................................................   1     1   seq
.....gggucggaguuagcucaagcggA..................................................................................   3     1   seq
.....ggUucggaguuagcucaagcggu..................................................................................   61    1   seq
.....gggucgAaguuagcucaagcggu..................................................................................   1     1   seq
.....gggucggaguuagcucaagcCgu..................................................................................   3     1   seq
.....gggucggGguuagcucaagcggu..................................................................................   1     1   seq
.....gggucggCguuagcucaagcggu..................................................................................   4     1   seq
.....gggucggaguuagcucaagcUgu..................................................................................   3     1   seq
.....gggucggaguuUgcucaagcggu..................................................................................   1     1   seq
.....ggUucggaguuagcucaagcgguu.................................................................................   24    1   seq
.....gggucggaguuagcucaCgcgguu.................................................................................   1     1   seq
.....gAgucggaguuagcucaagcgguu.................................................................................   1     1   seq
.....Aggucggaguuagcucaagcgguu.................................................................................   3     1   seq
.....gCgucggaguuagcucaagcgguu.................................................................................   3     1   seq
.....Cggucggaguuagcucaagcgguu.................................................................................   1     1   seq
.....gggucggaguuagcucaGgcgguu.................................................................................   2     1   seq
.....gggucggaguuagcucUagcgguu.................................................................................   1     1   seq
.....gggucggaguuagcucaagcgguu.................................................................................   167   0   seq
.....ggAucggaguuagcucaagcgguu.................................................................................   1     1   seq
.....gggucggaguuagcucaagcgUuu.................................................................................   1     1   seq
.....ggCucggaguuagcucaagcgguu.................................................................................   6     1   seq
.....gggucggaguuagcucaagcgguA.................................................................................   38    1   seq
.....gggCcggaguuagcucaagcgguu.................................................................................   1     1   seq
.....gggucggaguuagcucaaUcgguu.................................................................................   2     1   seq
.....ggguUggaguuagcucaagcgguu.................................................................................   1     1   seq
.....gggucggaguCagcucaagcgguu.................................................................................   1     1   seq
.....Uggucggaguuagcucaagcgguu.................................................................................   10    1   seq
.....gggucggaguuagcucaagcgguG.................................................................................   1     1   seq
.....gggucggaguuagcucaagcgguAa................................................................................   3     1   seq
.....gggucggaguCagcucaagcgguua................................................................................   1     1   seq
.....gggucggaguuagcucaagcgguuU................................................................................   55    1   seq
.....gggucggaguuagcucaagcgguua................................................................................   6     0   seq
.....Cggucggaguuagcucaagcgguua................................................................................   1     1   seq
.....gggucggaguuagcucaagcgguuac...............................................................................   3     0   seq
.....gggucggaguuagcucaagcgguuacc..............................................................................   8     0   seq
.....gggucggaguuUgcucaagcgguuacc..............................................................................   1     1   seq
.....ggUucggaguuagcucaagcgguuacc..............................................................................   1     1   seq
.....gggucggaguuagcucaagcgguuaccu.............................................................................   1     0   seq
......ggucggaguuagcucaagcgg...................................................................................   1     0   seq
......ggGcggaguuagcucaagcggu..................................................................................   1     1   seq
......ggucggaguuagcucaagcggu..................................................................................   6     0   seq
......Ugucggaguuagcucaagcgguu.................................................................................   1     1   seq
.......gucggaguuagcucaagcggu..................................................................................   1     0   seq
......................agcgguuaccuccucaugccgga.................................................................   2     0   seq
..........................guuaccuccucaugccggacu...............................................................   1     0   seq
............................uaccuccucaugccggacu...............................................................   1     0   seq
............................uaccuccucaugccggacuu..............................................................   5     0   seq
............................uaccuccucaugccgUacuu..............................................................   1     1   seq
............................uaccuccucaugccggacuuA.............................................................   1     1   seq
............................uaUcuccucaugccggacuuu.............................................................   1     1   seq
............................uaccuccucaugccggacuuu.............................................................   7     0   seq
............................uaGcuccucaugccggacuuu.............................................................   1     1   seq
............................uCccuccucaugccggacuuu.............................................................   1     1   seq
```

```
                                                                               hsa-miR-886-3p
   hsa-miR-886-5p
uacccgggucggaguuagcucaagcgguuaccuccucaugccggacuuucuaucuguccaucucugugcugggguucgagacccgcgggugcuuacugacccuuuuaugc

............................uaccuccucaugccggCcuuu............................................................. 1 1 seq
............................uaccuUcucaugccggacuuuc............................................................ 1 1 seq
............................uaccuccucaugccggacuuuc............................................................ 22 0 seq
............................uaGcuccucaugccggacuuucu........................................................... 2 1 seq
............................uaccuccucaugccUgacuuucu........................................................... 1 1 seq
............................uaccuccucCugccggacuuucu........................................................... 1 1 seq
............................uaccuccucaugccggacuuucu........................................................... 24 0 seq
............................uaccGccucaugccggacuuucu........................................................... 3 1 seq
............................uaccuccucaugccggacuuucua.......................................................... 4 0 seq
............................uaccuccucaugccUgacuuucuauc....................................................... 1 1 seq
............................uaccuccucaugccggacuuucuauc....................................................... 1 0 seq
..............................ccuccucaugccggacuuucu........................................................... 1 0 seq
...............................cuccAcaugccggacuuu............................................................. 1 1 seq
..................................cucaugccggacGuucuau......................................................... 1 1 seq
..................................cucaugccggacuuucuauc........................................................ 1 0 seq
..................................cucaGgccggacuuucuaucuguc.................................................... 1 1 seq
...................................uAaugccggacuuucuau......................................................... 1 1 seq
...................................ucaugccggacuuucuau......................................................... 1 0 seq
...................................ucaugccggacuuucuauG........................................................ 1 1 seq
...................................ucaugccggacuuucuaucugu..................................................... 1 0 seq
...................................ucaugccggacuuucuaucuguc.................................................... 1 0 seq
...................................ucaugccggacuuucuaucuguA.................................................... 1 1 seq
...................................ucaugccggacuuucuaucugucc................................................... 3 0 seq
....................................caugccggacuuucuaucugucc................................................... 1 0 seq
....................................caugccggacuuucuaAcugucc................................................... 1 1 seq
.....................................augccggacuuucuaucugucc................................................... 2 0 seq
.....................................Cugccggacuuucuaucugucc................................................... 1 1 seq
.....................................................cuguccaucucugugcugggU.................................... 1 1 seq
.......................................................guccaucucugugcuggggguucgaUac............................ 1 1 seq
.............................................................cucugugcugggguucgagacc........................... 2 0 seq
...............................................................cuUugcugggguucgagacc........................... 1 1 seq
.....................................................................ugggguucgagacccgcgggu.................... 1 0 seq
.......................................................................ggguucgagacccgcgggugcuua............... 1 0 seq
................................................................................acccgcgggugcuuacugacccuuu..... 2 0 seq
.................................................................................cccgcgggugcuuacugac.......... 1 0 seq
.................................................................................cccgcgggugcuuacugacccuuuA.... 1 1 seq
.................................................................................cccgcgggugcuuacugacccuuuu.... 1 0 seq
...................................................................................cgcgggugcuuacugacc......... 2 0 seq
...................................................................................cgcgggugcuuacuUacc......... 1 1 seq
...................................................................................cgcgggugcuuacugaccA........ 1 1 seq
...................................................................................cgcgggugcuuacugaccc........ 2 0 seq
...................................................................................cgcUggugcuuacugaccc........ 1 1 seq
...................................................................................cgcgggugcuuacugacccu....... 3 0 seq
...................................................................................cgcgggugcuGacugacccuu...... 1 1 seq
...................................................................................cgcgCgugcuuacugacccuu...... 1 1 seq
...................................................................................cgcgggugcuuCcugacccuu...... 1 1 seq
...................................................................................cgcgggugcuuacugacccuu...... 18 0 seq
...................................................................................cgcgggAgcuuacugacccuu...... 1 1 seq
...................................................................................cgcggguUcuuacugacccuuu..... 1 1 seq
...................................................................................cgcgggGgcuuacugacccuuu..... 1 1 seq
...................................................................................cgcgggCgcuuacugacccuuu..... 1 1 seq
...................................................................................cgcgggugcuuacugacccuuu..... 205 0 seq
...................................................................................cgcgggugcuuacugacccuGu..... 1 1 seq
...................................................................................cgcgCgugcuuacugacccuuu..... 2 1 seq
...................................................................................cgcggCugcuuacugacccuuu..... 1 1 seq
...................................................................................cgcgggugcuuacugacccuuA..... 5 1 seq
...................................................................................cgcgUgugcuuacugacccuuu..... 3 1 seq
...................................................................................cgUgggugcuuacugacccuuu..... 1 1 seq
...................................................................................cgcgggugcuuCcugacccuuu..... 2 1 seq
...................................................................................cgcgggugcuuacugCcccuuu..... 1 1 seq
...................................................................................cgcgggugcuuacugacccuuuA.... 2 1 seq
...................................................................................cgcggguUcuuacugacccuuuu.... 1 1 seq
...................................................................................cgcUggugcuuacugacccuuuu.... 1 1 seq
...................................................................................cgcgggugcuuacugacccuuuu.... 10 0 seq
...................................................................................cgcgggugcuuacugacccuuuGa... 1 1 seq
....................................................................................gcgggugcuuacugacccuuu..... 1 0 seq
.......................................................................................ggugcuuacugacccuuuuU... 1 1 seq
........................................................................................guUcuuacugacccuuuu.... 1 1 seq
........................................................................................gugcuuacugacccuuuu.... 1 0 seq
```

hsa-miR-886-3p

hsa-miR-886-5p

uacccgggucggaguuagcucaagcgguuaccuccucaugccggacuuucuaucuguccaucucugugcuggggguucgagacccgcgggugcuuacugacccuuuuaugc
